# A Fully Integrated Closed‐Loop System Based on Mesoporous Microneedles‐Iontophoresis for Diabetes Treatment

**DOI:** 10.1002/advs.202100827

**Published:** 2021-06-03

**Authors:** Xiangling Li, Xinshuo Huang, Jingshan Mo, Hao Wang, Qiqi Huang, Cheng Yang, Tao Zhang, Hui‐Jiuan Chen, Tian Hang, Fanmao Liu, Lelun Jiang, Qianni Wu, Hongbo Li, Ning Hu, Xi Xie

**Affiliations:** ^1^ The First Affiliated Hospital of Sun Yat‐Sen University State Key Laboratory of Optoelectronic Materials and Technologies School of Electronics and Information Technology; Guangdong Province Key Laboratory of Display Material and Technology Sun Yat‐Sen University Guangzhou China; ^2^ School of Biomedical Engineering Sun Yat‐SenUniversity Guangzhou China; ^3^ Zhongshan Ophthalmic Center Sun Yat‐Sen University Guangzhou China

**Keywords:** mesoporous microneedles‐iontophoresis, minimally invasive, diabetes monitor and therapy, intelligent wearable device, closed‐loop system

## Abstract

A closed‐loop system that can mini‐invasively track blood glucose and intelligently treat diabetes is in great demand for modern medicine, yet it remains challenging to realize. Microneedles technologies have recently emerged as powerful tools for transdermal applications with inherent painlessness and biosafety. In this work, for the first time to the authors' knowledge, a fully integrated wearable closed‐loop system (IWCS) based on mini‐invasive microneedle platform is developed for in situ diabetic sensing and treatment. The IWCS consists of three connected modules: 1) a mesoporous microneedle‐reverse iontophoretic glucose sensor; 2) a flexible printed circuit board as integrated and control; and 3) a microneedle‐iontophoretic insulin delivery component. As the key component, mesoporous microneedles enable the painless penetration of stratum corneum, implementing subcutaneous substance exchange. The coupling with iontophoresis significantly enhances glucose extraction and insulin delivery and enables electrical control. This IWCS is demonstrated to accurately monitor glucose fluctuations, and responsively deliver insulin to regulate hyperglycemia in diabetic rat model. The painless microneedles and wearable design endows this IWCS as a highly promising platform to improve the therapies of diabetic patients.

## Graphical abstract

A fully integrated wearable closed‐loop system (IWCS) based on microneedle‐iontophoresis platform is developed for in situ diabetic sensing and treatment. This IWCS is demonstrated to accurately monitor glucose fluctuations and responsively deliver insulin to regulate hyperglycemia. The painless microneedles and wearable design endow this IWCS as a highly promising platform to improve the therapies of diabetic patients.

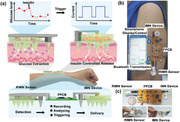

## 1 Introduction

Smart point‐of‐care electrical systems that can monitor disease and provide intelligent therapies are highly promising for the emerging development of precision medicine.^[^ ^1^ ^]^ Diabetes mellitus, a common metabolic disorder that has threatened 463 million people worldwide, could severely affect the patients' health qualities and lead to painful complications of cardiovascular, renal disease, and neurodegeneration.^[^ ^2^ ^]^ At present, the traditional diabetes therapy has been relying on blood glucose (BG) measurement using finger prick, followed by manual injections of insulin to regulate the hyperglycemia.^[^ ^3^ ^]^ However, these traditional sampling and injection methods are generally accompanied by pains, inconvenience, and potential infections, and fail to continuously track BG fluctuations and timely regulate diabetes. A closed‐loop system that could automatically monitor BG and respond rapidly to treat diabetes would be in line with the megatrend of smart medicine and improve the life quality of patients.^[^ ^4^ ^]^

Over the past few decades, invasive continuous glucose monitors (CGMs) based on implantable electrodes were commercialized as sophisticated biosensors for glycemic tracking in subcutaneous interstitial fluid with clinically satisfying accuracy.^[^ ^5^ ^]^ Especially when coupled with automatic insulin pump, the CGM technology could potentially offer a fully intelligent approach for regulating diabetes, alleviating the stress of self‐management.^[^ ^6^ ^]^ Despite of the current success, the long feature of implanted CGM electrodes or insulin pump tubing could often lead to undesirable pain, bleeding, and inflammation, as well as interference to life activities.^[^ ^7^ ^]^ On the other hand, non‐invasive wearable glucose sensors including bracelets,^[^ ^8^ ^]^ contact lenses,^[^ ^9^ ^]^ and sweat‐based sensors have attracted increasing research interests.^[^ ^10^ ^]^ However, non‐invasive sensors are rarely able to accurately reflect and regulate glucose levels, since the attempts to avoid skin penetration result in insufficient access to the glucose in blood or interstitial fluids in vivo.^[^ ^11^ ^]^ Iontophoretic technologies have recently been integrated with wearable devices to electrically control and enhance the permeation of glucose or drug molecules across stratum corneum.^[^ ^10^, ^12^ ^]^ However, glucose measurements by reverse iontophoresis alone hardly meets the clinically required accuracy due to the barrier of stratum corneum.^[^ ^13^ ^]^ In addition, transdermal drug deliveries by iontophoresis are often limited to small molecules, while permeation of large molecules such as insulin are less compatible with iontophoresis.^[^ ^14^ ^]^

Recently, microneedles (MNs) with controlled length of 500–800 µm have been demonstrated as highly promising tools for transdermal biosensing or drug delivery, since the MNs could penetrate stratum corneum without causing bleeding or pain by avoiding damage to the peripheral nerves or capillaries.^[^ ^15^ ^]^ Especially for diabetes managements, biosensors based on hollow MNs array^[^ ^16^ ^]^ and solid MNs^[^ ^17^ ^]^ have been developed to detect glucose in vivo, while insulin‐loaded degradable^[^ ^18^ ^]^ or hollow MN patches^[^ ^19^ ^]^ have also been demonstrated to treat hyperglycemia effectively. While most of the existing MN platforms focus on sensing or delivery separately, MNs‐based integration systems for closed‐loop diabetic management remain undeveloped. This dilemma is mostly due to the complicated micro‐fabrication process of miniature MN devices, and the critical requirements of accurate sensing and controlled delivery.^[^ ^20^ ^]^ Until recently, Lee et al. demonstrated the concept of sweat glucose sensor based on non‐invasive wearable design, connecting to a dissolvable MN patch for heat‐triggered metformin release.^[^ ^21^ ^]^ To detect actual glucose in vivo that could fit better to clinical applications, Gu et al. developed advanced chemical strategies using passive microneedles to mediate transdermal implantation of smart nano‐vesicles, which could rapidly release insulin in response to glucose levels.^[^ ^2^, ^22^ ^]^ To achieve real‐time recording and display of glucose level for clinical diagnosis and tracking, MN platforms based on integrated electrical devices would still be more desirable, yet they have not been realized.

**Figure 1:**
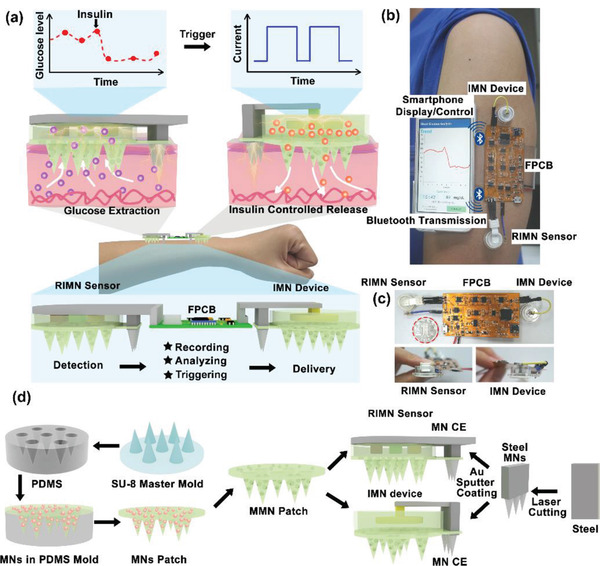
a) Illustration of the IWCS based on MN platform for real‐time and in situ diabetes monitoring and treatment. The IWCS consisted of a RIMN glucose sensor, a FPCB, and IMN therapeutic components. The system detected glucose fluctuation via the RIMN, where the signals were recorded, transmitted, and had given feedback via FPCB, triggering release of insulin via IMN device. b) The photographic image showing the concept of IWCS worn on human arm and wireless communication with smartphone for data transmission and display. c) The digital photos of IWCS, including zoom‐in image of RIMN sensor (left) and MN device (right), where a coin (marked with red circle) for visually size comparison. d) Illustration of the fabrication procedure of the whole IWCS and each component. The essential component, MMN patch, was fabricated via a typical molding technique, coupled with template perforation and porogen removal. The MMN and iontophoretic electrodes were assembled by a 3D‐printed chamber to form the RIMN Sensor and IMN device.

Herein, for the first time to our knowledge, a fully integrated wearable closed‐loop system (IWCS) based on MN platform was developed for real‐time and in situ diabetes monitoring and treatment (**Figure** **1**a). In our design, the key component was mesoporous microneedles (MMN) patches, which served as the tool for penetrating the stratum corneum in a pain‐free manner, implementing subcutaneous substance exchange for both glucose extraction and insulin delivery. When MMN arrays were coupled with either reverse iontophoresis or iontophoresis, glucose extraction or insulin delivery could be significantly enhanced and become electrically tunable compared to free diffusion. The IWCS consisted of three connected modules: 1) a MN glucose sensor (based on MMN‐reverse iontophoretic extraction and electrochemical sensing); 2) a flexible printed circuit board (FPCB) as integrated recording and control section; and 3) a MN therapeutic component (MMN‐iontophoretic insulin delivery by electrical trigger). This IWCS was demonstrated to accurately track glucose fluctuations, and responsively release insulin for effective regulation of BG in a diabetic rat model. The minimally invasive nature of MMN and wearable design endowed our intelligent system as a highly promising platform for facilitating diabetes therapies and improving life quality of patients.

## 2 Results and Discussion

### 2.1 Materials Fabrication and Device Design

The whole system was designed to be compact and wearable on the human arm, and sufficiently intelligent by electrical recording and control. In the RIMN sensor, the glucose in interstitial fluids was extracted into the sensor chamber via reverse iontophoresis after the MN pierced the stratum corneum, followed by electrochemical detection via a three electrodes system. The flexible circuit module processed the measured glucose signals and transmitted wirelessly to an external smartphone via Bluetooth communication (Figure 1b). Control signals fed back by the FPCB triggered the IMN device to deliver insulin via iontophoresis at hyperglycemia condition (>200 mg dL^−1^). The IWCS possessed the following advantages: 1) painless and minimally invasive to skin due to the use of MN structure; 2) sufficient access to subcutaneous fluid due to the mesoporous feature of MMN; 3) enhanced glucose extraction for sensing and electrically controllable insulin delivery due to the coupling with iontophoretic techniques; 4) in situ and real‐time monitoring and therapy due to wearable and compact design; and 5) data trackable, highly intelligent, fast responsive, and quantitative management due to wireless communications with smartphone (Figure 1c and Figure S1, Supporting Information).

The MMN patch was fabricated via a typical molding technique, coupled with template perforation and porogen removal method to produce nanopores in MN (Figure 1d). Briefly, a micro‐fabricated SU‐8 MN patch using lithography was employed as the master mold to generate the inverted polydimethylsiloxane (PDMS) mold. Mixture of poly (glycidyl methacrylate) (PGMA) and polyethylene glycol (PEG) as porogen in 2‐methoxyethanol was centrifugated into the PDMS mold, where the PGMA was crosslinked (Irgacure 184 as photoinitiator) under ultraviolet light to produce the MN structure. The as‐fabricated MN patch were separated from the PDMS mold, followed by dissolving the porogen of MN patch in methanol/water (1:1) overnight to form mesoporous morphology. On the other hand, metal MN fabricated by laser micromachining from stainless steel plates was sputtered with Au and utilized as C.E. for both iontophoresis and reverse iontophoresis. The RIMN sensor consists of four components: 1) the MMN for accessing interstitial fluid; 2) a planar glucose electrode (three‐electrode system) for detection of extracted glucose; and 3) a reverse‐iontophoresis extraction system (Ag/AgCl working electrode and MN C.E.) to enhance glucose extraction, and a 3D printed chamber for sensor integration. The IMN delivery device included three parts: 1) the MMN for penetrating skin; 2) an iontophoresis system (a planar working electrode and a MN C.E.) for controlling insulin release; and 3) a 3D printed chamber for device integration (Figures S2 and S3, Supporting Information). The FPCB was connected to both RIMN sensors and IMN devices to create miniature IWCS that can be attached to the human arm.

**Figure 2:**
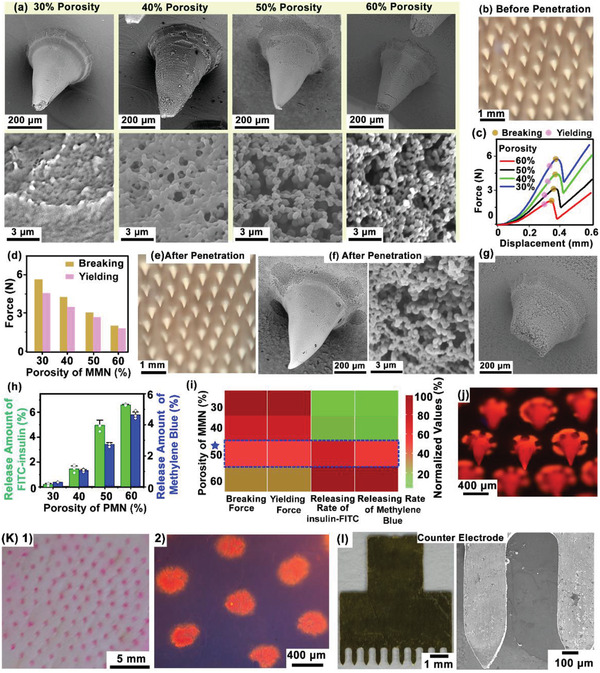
Characterizations of MMN. a) The SEM images showing a series of MMN with different porosities (30–60%). b) Digital photograph showing the morphology of MMN patch. c) The stress–strain test of MMN by dynamometer. The critical breaking and yielding forces were labeled, respectively. d) Quantitative analysis of the critical breaking and yielding forces of MMN with different porosities. e,f) Optical and SEM images showing MMN with 50% porosity could remain intact after insertion into skin. g) SEM image showing mechanical failure of MMN with 60% porosity after insertion into skin. h) Quantification of the molecular (methylene blue and insulin‐FITC) diffusion rates across the interface of MMNs with different porosities. *N* = 3 measurements. i) Heatmap plot summarizing the behaviors of the tested mechanical failure forces (yielding and breaking) and the diffusion rates (methylene blue and insulin‐FITC) at different MN porosities. j) Fluorescence images of MMN stained with Rhodamine B. k) Photograph and fluorescence image showing Rhodamine B deposition into pigskin by MMN penetration. l) Optical photograph and SEM images of MN C.E.

The MN distributed homogenously on a patch (≈4.3 cm^2^), with a needle density of 31.8 needles/cm^2^. Each needle possessed a conical structure, with ≈600 µm length and 400 µm base‐diameter, as visualized by scanning electron microscopy (SEM) and optical microscopy (**Figure** **2**a,b and Figure S4, Supporting Information). This specific length would enable MN to penetrate the stratum corneum but not reach to nerves or blood vessels in the dermis to cause pain or bleeding.^[^ ^15a^ ^]^ The porosity of the MMN could be tuned from 30–60% by adjusting the ratio of PEG porogen during fabrication process. The surface morphology on MN was characterized via SEM, where the mesoporous structure could be obviously observed when the porosities exceeded 40% (Figure 2a). The higher porosity of MNs would facilitate glucose extraction and drug delivery, but the mechanical durability was compromised. The stress–strain behavior of MN with different porosities (30–60%) was tested with a dynamometer (Figure S5, Supporting Information) and quantitatively analyzed for the mechanical failure forces (Figure 2c). The critical breaking and yielding forces are labeled in Figure 2d, which decreased with the increase of porosities. The structure and surface morphology of MMN with 50% porosity (≈2.8 n yielding force and ≈3.1 n breaking force) could remain intact after insertion into skin(Figure 2e,f), while the MMN with 60% porosity (≈1.6 n yielding force and ≈1.9 n breaking force) were prone to fracture after insertion into pig skin (Figure 2g).

The molecule diffusion rates across MMN with different porosities were examined using methylene blue (*M*_w_: 319.8, glucose substitute, 1 mg mL^−1^) and insulin‐FITC (*M*_w_: 6122.9, 5 mg mL^−1^). Solution containing either methylene blue or insulin‐FITC was dropped onto the backside of the MMN patch, allowing free diffusion to the PBS solution at the frontal side of MN tips (Figure S6, Supporting Information). After diffusion for 1 h, the released amounts of molecules were quantitatively analyzed via light absorption measurements. The results suggested that the molecule diffusion rates of both insulin‐FITC and methylene blue increased with increasing porosities, where the molecular diffusion rates were highly limited when the porosity was less than 40% (Figure 2h). In order to optimize the porosity, the values of the tested mechanical failure forces (yielding and breaking) and the diffusion rates (methylene blue and insulin‐FITC) at different MN porosities were normalized and quantitatively compared in a heatmap plot (Figure 2i). Based on the results, the MNs with porosity of 50% exhibited both satisfying mechanical behavior and molecule diffusion rates, and thus was selected as the optimized MN structure for constructing both RIMN sensors and IMN device in this work. To demonstrate MN penetration into skin, the MMN patch was stained with red fluorescent dye (Rhodamine B) followed by observation of fluorescence dye deposition into skin after penetration. After MN were pressed against porcine skin for 5 min and then withdrawn, red fluorescent spots with similar size to MN diameter were observed in the skin, aligning as array corresponding to the MN geometry. Magnified fluorescence images revealed the detailed deposition profile of red fluorescent dye in the skin, confirming MMN penetration into the subcutaneous space (Figure 2j,k and Figure S7, Supporting Information). Meanwhile, laser micromachining was employed to fabricate a 2D‐metal MN patch from a stainless steel‐plate. The metal MN was designed to possess ≈225 µm‐diameter at the base, ≈800 µm‐length, and ≈250 µm interval between adjacent MNs (Figure 2l). The steel MN was then sputtered with ≈100 nm Au layer to enhance biocompatibility, which was employed as C.E. for both iontophoresis and reverse iontophoresis in the IWCS.

### 2.2 Performance and Characterization of RIMN Sensor

**Figure 3:**
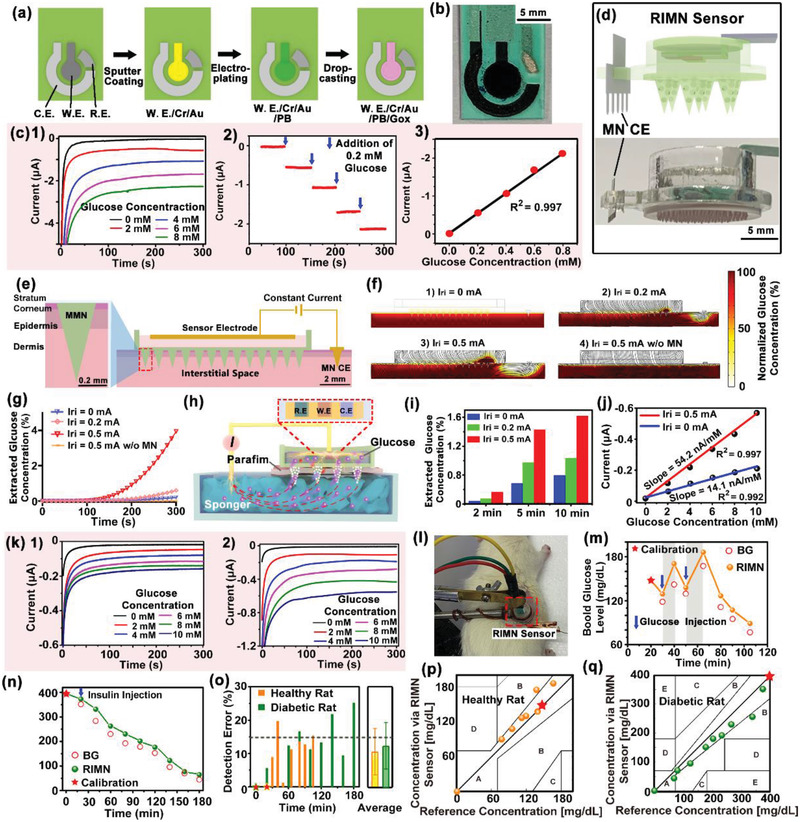
Performance characterization of the RIMN sensor. a) Illustration of the fabrication procedure and b) the optical image of planar glucose electrode. c) The amperometric response of planar glucose electrode. 1) The electrode was tested with a series of glucose solution (0–0.8 mm). 2) Glucose concentration stepwise‐increased by 0.2 mm (blue arrows). 3) The linear relations of the current signals and the corresponding glucose concentrations. d) Schematic diagram (top) and optical image (bottom) of the RIMN sensor. e) COMSOL 2D model of glucose extraction by RIMN. f) The electrical field distribution (indicated with black curves) and glucose concentration profile (indicated with brown color) at *t* = 300 s after reverse iontophoresis. g) Concentration–time relations of subcutaneous glucose extraction were quantitatively analyzed. h) Schematic showing the experimental setup of RIMN sensor for glucose sensing in vitro. i) Quantification of glucose extraction efficiency under different iontophoretic currents and time conditions. j) The linear relations of the current signals and glucose concentrations via RIMN sensors. k) The amperometric responses of RIMN sensor upon different glucose concentrations under 0 and 0.5 mA reverse iontophoretic extraction for 5 min. l) Photograph showing the application of RIMN sensor on anesthetized rat. m,n) The dynamic glucose signals recorded by RIMN sensors on m) healthy rat and n) diabetic rat model. The detected current signal via RIMN was converted to glucose concentration and the actual BG were measured via standard glucose test strips. The asterisk indicated the calibration point. The blue arrow indicated the time point of glucose or insulin injections. o) Statistical analysis showing the detection errors of RIMN sensor compared to the actual BG at corresponding time points. The asterisks indicated calibration points. The dash line indicated the clinical criterion of error <15%. p,q) Clarke's error grid analysis showing the detection accuracy of RIMN sensor compared to the actual BG. The asterisks indicated calibration points.

MMNs were employed for constructing the RIMN sensor. When coupled with reverse iontophoresis, the glucose in interstitial fluid would diffuse through the mesoporous tip channels of MN to the sensing chamber at the backside of patch. The glucose detection employed the standard amperometric strategy of an enzymatic three‐electrodes electrochemical system, including an enzymatically functionalized carbon electrode as W.E., a Pt‐plated carbon electrode as C.E., and an Ag/AgCl electrode as R.E. The fabrication procedure of planar glucose electrode supported on plastic substrate is illustrated in **Figure** **3**a,b and Figure S8a, Supporting Information. At the W.E., 50 nm‐thick Cr (as metal adhesion layer) and 80 nm‐thick Au layers were deposited on screen‐printed carbon electrode by magnetron sputtering. Prussian blue (PB) was then electrodeposited in situ on the W.E., followed by immobilization of glucose oxidase on the surface by crosslinking with bovine serum albumin and glutaraldehyde (Figure S8b,c, Supporting Information). In principle, in the presence of glucose oxidase (GO*_x_*) as enzyme, glucose in solution can be selectively oxidized into gluconic acid and hydrogen peroxide (H_2_O_2_) by the following reactions:

(1) $$\mathrm{Glucose}+H_{2}O+O_{2}\;\overset{GO_{x}}{\to }\;\mathrm{Gluconic}\;\mathrm{acid}+H_{2}O_{2}$$

H_2_O_2_ acts as an oxidizing agent to release free electrons crossing conductor and produces an electrical response, where the glucose concentration can be identified by the current response. In order to reduce polarization on metal electrode, redox‐active material such as PB was electrochemically coated on the Au electrode to provide better selectivity and sensitivity.^[^ ^23^ ^]^ More importantly, since the reduction potential of PB in glucose solution is close to 0 V referenced to Ag/AgCl, it is possible to eliminate signal interference by the oxidation of other components (e.g., ascorbic acid, uric acid, and lactic acid) at higher bias potentials.^[^ ^11b^ ^]^

The response of as‐fabricated electrode was tested by challenging with a series of concentration gradients (0–0.8 mm) of glucose concentrations. The current increased by ≈219 nA mm ^−1^ immediately after addition of glucose concentration and reached steady‐state current (Figure 3c). Considering the glucose in interstitial fluid would be extracted through MMN into the backside chamber and gets diluted, the sensing linear region of the bare glucose electrode was tuned to be 0–1 mm, so that the final MN‐integrated glucose sensor could possess sensing linear region in the range of 0–20 mm to meet diabetic applications. The mesoporous structure of MN served essentially as an effective layer to tune the sensitivities and detection range of the glucose sensor.

The planar glucose electrode was covered on top of the MMN backside, and the gap between electrode surface and MMN substrate was filled with PBS solution. The MMN and glucose electrode were assembled with a metal iontophoretic MN C.E. by a 3D‐printed chamber (12 mm‐diameter × 5 mm‐height) to form the RIMN Sensor (Figure 3d and Figure S3, Supporting Information). Theoretical simulation via COMSOL Multiphysics 5.5 was employed to evaluate the mechanism of transdermal glucose extraction by the RIMN sensor. The electric currents interface and the transport of diluted species interface of COMSOL Multiphysics enable the calculation of an electrically‐driven glucose diffusion profile (Figures S9 and S10, Supporting Information). The simulation used a simplified 2D model as illustrated in Figure 3e, with the geometries and components mimicking the actual setup. The skin tissue was modeled as three layers (stratum corneum, epidermis, and dermis) with corresponding mass diffusivities and electric conductivities. MMN of RIMN sensor was inserted into the skin. A sensor electrode on top of MMN for reverse iontophoresis, coupled with a MN C.E. placed next to it. Glucose was modeled with a constant concentration source at the bottom area of dermis, and the accumulative amount of glucose underneath in the sensing chamber after reverse iontophoretic extraction was calculated. The time slots of electrical field distribution (indicated with black curves) and a glucose concentration profile (indicated with brown color) after reverse iontophoresis at 0, 0.2, and 0.5 mA are plotted as in Figure S11, Supporting Information (the typical profile at *t* = 300 s is shown in Figure 3f). The glucose gradually passed through the MMN under free diffusion and electric field, where the extracted glucose increased with extraction time and with the enhancement of iontophoretic currents (Iri). As a control, glucose extraction by reverse iontophoresis using planar electrode without MMN was 452‐folds (300 s‐reverse iontophoresis) lower than the RIMN, due to the difficulty of glucose diffusion across the stratum corneum. The results also showed that the enhanced of MMN porosities (testing from 30% to 60%) would significantly improve the glucose extraction (Figure 3g).

To confirm the theoretical calculation, glucose sensing using RIMN sensor in vitro was investigated. The setup of artificial skin tissue was constructed using a sponge (70% porosity) filled with glucose solution, where a water‐impermeable parafilm (≈100 um thickness) was placed on top of the sponge to mimic the stratum corneum.^[^ ^18b^ ^]^ The RIMN sensor was placed above the parafilm, with the MMN penetration through the parafilm (Figure 3h). The concentrations of the extracted glucose in the sensor chamber at different iontophoretic currents (0, 0.2, and 0.5 mA) and different iontophoresis durations (2, 5, and 10 min) were quantitatively measured by glucose assay kit (Figure S14, Supporting Information). The results showed that the amount of glucose extraction for 5 min was 5.5‐folds higher than that for 2 min, but was not significantly lower compared to the extraction for 10 min. On the other hand, glucose extraction via 0.5 mA iontophoretic current was enhanced by 1.8‐folds than 0.2 mA, and enhanced by 3‐folds than free diffusion (Figure 3i). This suggested that reverse iontophoresis could effectively recruit glucose for enhancing sensing, and 0.5 mA current and 300 s duration could be employed as the optimized iontophoretic condition. The in‐situ detection of glucose with different concentrations (2–10 mm, the typical magnitude of glucose concentration in interstitial fluid) in vitro^[^ ^12a^ ^]^ by the RIMN sensor was further investigated. The detection signal increased with the increase of glucose concentration in a linear trend, which could be further employed as standard curve for converting current signal to glucose concentration. As shown in Figure 3j,k, the detection sensitivity was 54.2 nA mm ^−1^, which was ≈3.8‐folds higher than then sensitivity (14.1 nA mm ^−1^) of MMN sensor without reverse iontophoresis (0 mA). These results demonstrated that the as‐constructed RIMN sensor could detect glucose with satisfying sensitivity and detection range for diabetic applications.

In situ and real‐time glucose monitor using RIMN sensor on live animals was further demonstrated. The hair on the back of an anesthetized healthy Sprague‐Dawley (SD) rat was shaken, and the RIMN sensor was pressed against the back to penetrate the skin by MMN (Figure 3l). Glucose sensing was performed every 10 min at the iontophoretic condition of 0.5 mA current and 300 s duration for each cycle. The rat was injected intraperitoneally with 5% glucose solution at time points *t* = 30 min and *t* = 50 min (indicated by blue arrows in Figure 3m, the process in Figure S15, Supporting Information), in order to induce fluctuation of glucose levels in vivo for sensor evaluation. In a separated experiment, the RIMN sensor was performed on a streptozotocin‐induced diabetic rats, with subcutaneous injection of 5 UI insulin at *t* = 20 min (indicated by blue arrows in Figure 3n, the process in Figure S16, Supporting Information). To examine the detection accuracy of the RIMN sensor, the BG of rat (red hollow point in Figure 3m,n) was measured every ≈10 min using standard glucose test strips method as positive control. The detected current signal via RIMN was converted to glucose concentration according to the in vitro standard curve of RIMN sensor. Considering the difference between in vivo environment and in vitro environment, the glucose detection results in vivo via RIMN were calibrated using the first‐measured BG data point (at *t* = 20 min in healthy rat, *t* = 0 min in diabetic rat, indicated with red star). The glucose concentration of the healthy rat was observed to increase over a short period (10 min, indicated by the gray band in Figure 3m) right after glucose injection, while the blood glucose of the diabetic rat rapidly dropped below 200 mg dL^−1^ within an hour after the subcutaneous injection of insulin. The RIMN could track the glucose fluctuation in a similar profile of standard BG measurements, where the accuracies were quantified and examined with Clarke's error grid analysis. The error of all the RIMN‐measured glucose signal was below 25%, with an average error of 10.4 ± 6.4% for the healthy group and 12.9 ± 6.9% for the diabetic group (Figure 3o). In the Clarke error grid analysis, 87.5% of healthy group data and 90% of diabetic group data located in region A, corresponding to a sensing error <20% that is closely satisfying the clinical requirement of error <15% (Figure 3p,q). Uses of more calibration points could further improve the sensing accuracy, but induce more pain and inconvenience due to frequent BG sampling (Figures S17 and S18, Supporting Information). In addition, although there is a lag time of ≈5 min between the changes in interstitial glucose concentration and plasma, it could be calibrated by algorithm in actual applications. These results indicated the RIMN sensor could be applied for in situ glucose monitor on animals with satisfying accuracy and performance.

### 2.3 Performance and Characterization of IMN Device

#### 2.3.1 In Vitro Studies of the IMN Device for Transdermal Insulin Release

**Figure 4:**
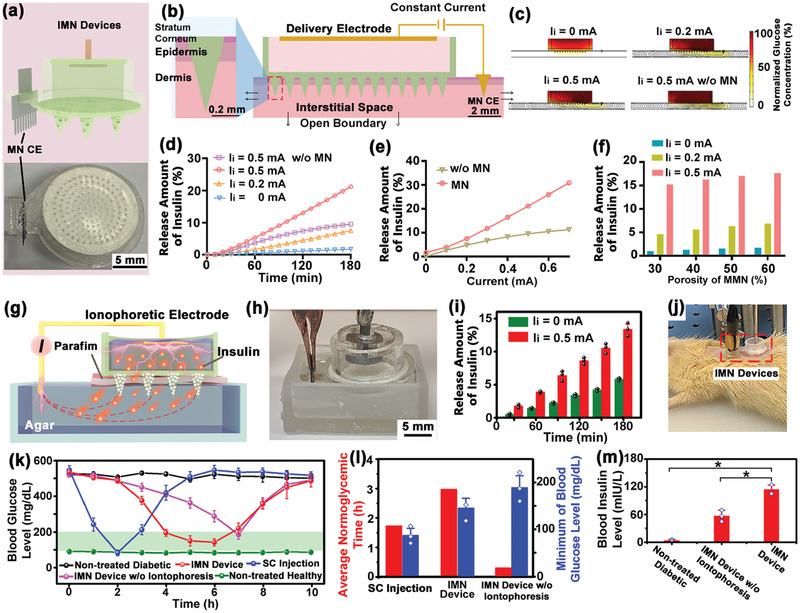
Performance characterization of the IMN Device. a) The schematic diagram (top) and optical image (bottom) of IMN device. b) Illustration of the COMSOL 2D model of IMN device for transdermal insulin delivery. c) The electrical field distribution (indicated with black curves) and insulin concentration profile (indicated with brown color) after iontophoresis at 0, 0.2, 0.5 mA for *t* = 180 min via IMN device. Theoretical calculations of the effects of d) iontophoretic duration, e) electric filed, and f) MN porosity on delivery. g) Schematic and h) photographs showing the experimental setup of IMN device for insulin delivery in vitro. i) Quantification of insulin released from the IMN device for 180 min, either at constant iontophoretic current (0.5 mA) or free diffusion (0 mA). *N* = 3 measurements. j) Photograph showing application of IMN device on anesthetized rats. k) Diabetic rats were treated via IMN device, non‐iontophoretic MN device, and subcutaneous injection of insulin, while the non‐treated diabetic and healthy rats were used as controls. After treatments, the BG fluctuations were continuously monitored for 10 h. The green region indicated the normoglycemia. *N* = 3 BG measurements. l) Quantitative analysis of the corresponding durations at normoglycemia and the minimum BG for different treatments. *N* = 3 BG measurements. m) Measurement of the plasma insulin concentrations of diabetic rats treated with IMN device and non‐iontophoretic MN device for 2 h. *N* = 3 measurements. Data were presented as mean ± SD. Significance was calculated by one‐way ANOVA. *p <0.05.

MMN were also employed for constructing the IMN device, where the MMN mediated delivery of insulin from the backside chamber into the dermis in a painless manner. The free diffusion of insulin continuously into interstitial fluid through MMN formed a basal delivery to control the basic glucose level, while iontophoresis was coupled to facilitate a bolus delivery to minimize glucose fluctuation. An Au‐coated electrode was placed on top of the MMN, and the gap between electrode surface and MMN substrate was filled with an insulin solution‐loaded sponge. The free diffusion rate of insulin could also be retarded and tuned by a sponge with a certain porosity. The MMN, delivery electrode and a metal iontophoretic MN C.E. were assembled by a 3D‐printed chamber form the IMN device (**Figure** **4**a). Overall the delivery of insulin could be modulated by the loaded insulin concentration, the iontophoretic current, and durations. The insulin could also be flexibly supplemented into the device without limitations of drug loading doses. The mechanism of transdermal insulin administration by the IMN devices was theoretically simulated via the electric currents interface and the transport of diluted species interface of COMSOL Multiphysics, same as the simulation model of RIMN for glucose extraction (Figure S6.1, Supporting Information). The MMN was inserted into the skin with a pair of iontophoretic electrodes placed next to it (Figure 4b). Insulin was modeled with a constant concentration source in the internal chamber region and the accumulative amount of insulin in dermis after delivery was calculated. The time slot of electrical field distribution (indicated with black curves) and insulin concentration profile (indicated with brown color) after iontophoresis at 0, 0.2, and 0.5 mA are plotted as in Figures S20–S22, Supporting Information (the typical profile at *t* = 180 min is shown in Figure 4c). The effects of iontophoretic duration, electric filed, and MN porosity on delivery were systematically examined, as shown in Figure 4d,e. The insulin could gradually pass through the MMN under free diffusion, and be significantly facilitated by the iontophoretic current (Ii), for example, 12.3‐folds enhanced after 180 min at Ii = 5 mA (Figure 4d). The deliveries were improved with the increase of iontophoretic current and iontophoretic duration in a nearly linear profile. Without MN, calculation of insulin delivery by conventional iontophoresis with a pair of planar electrodes showed ≈2.2‐folds lower than the IMN, while in reality it would be more challenging to deliver insulin via conventional iontophoresis due to low skin permeability of insulin (Figure 4d). The calculation results also showed that the increase of MMN porosities (30% to 60%) would slightly enhance the insulin release (Figure 4e).

Next, insulin released using IMN device in vitro was investigated. As shown in Figure 4g,h, the setup of artificial skin tissue was constructed with a water‐impermeable parafilm (mimicking the stratum corneum) covered on top of an agar (1 wt%). Insulin solution (4 mg mL^−1^) released from the IMN device either at constant iontophoretic current (0.5 mA) or free diffusion, and the delivery amounts up to 180 min were quantitatively measured by a Coomassie Plus protein assay (Figure S23, Supporting Information). The IMN device released a small amount of insulin (5.8 ± 0.15% for 180 min) by free diffusion which formed the basal delivery. While iontophoretic current (0.5 mA) was coupled the delivery was enhanced by ≈2.3‐folds, suggesting a bolus delivery controlled by electrical field was feasible (Figure 4i).

To evaluate the efficacy of the treatment of diabetes via IMN device in vivo, different scenarios of insulin deliveries (Figure 4j) including using IMN device, non‐iontophoretic MN device, and subcutaneous injection of insulin were tested on anesthetized type 1 diabetic rats (induced by streptozotocin). Diabetic rats were administered by MN devices loaded with 4 mg mL^−1^ insulin solution for 3 h, while an equal amount of released insulin (≈5 IU) was directly subcutaneously injected as positive control. The BG of rats were dynamically tracked every ≈60 min using standard glucose test strips method (Figure 4k). The BG in the non‐treated healthy and diabetic groups were stable at 91.8 ± 4.3 and 531.6 ± 11.2 mg dL^−1^, respectively. The BG of rats treated by IMN device at 0.5 mA effectively dropped to normoglycemia (<200 mg dL^−1^) lasting for 3.3 h, with a minimum value of 148.2±20 mg dL^−1^ (Figure S24, Supporting Information). The BG in diabetic rats treated with subcutaneous injection decreased rapidly to a minimum of 88.8 ± 14.8 mg dL^−1^ at *t* = 2 h, but rebounded rapidly to the hyperglycemia state (>200 mg dL^−1^) with limited duration at normoglycemia (lasting for 1.7 h). The treatment with MN without iontophoresis almost failed to reduce the BG to normoglycemia (only lasting for 0.3 h with a minimum value of 191.4 ± 25 mg dL^−1^). The corresponding duration of glucose regulation at normoglycemia and the minimum BG was quantitatively compared (Figure 4l), where the IMN group showed advantages on both effectively reducing BG level and a longer normoglycemia duration. In addition, the plasma insulin concentrations of diabetic rats treated with the IMN device and non‐iontophoretic MN device for 2 h were increased to 113.8 ± 9.9 and 56.8 ± 12.4 mIU/L (Figure 4m), respectively, confirming that the iontophoresis could enhance transdermal delivery of insulin in vivo.

### 2.4 Integration of IWCS and Performance Characterization

**Figure 5:**
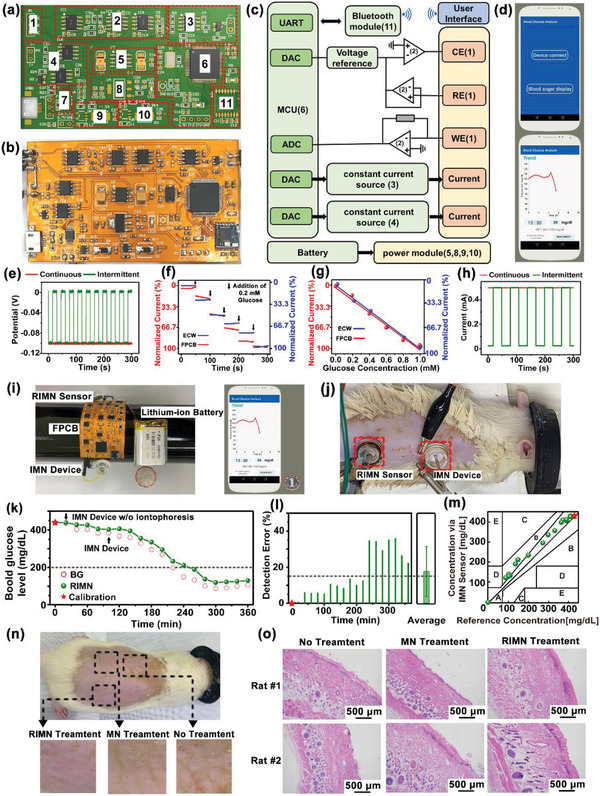
Performance characterization of the whole IWCS. a) Design sketch and b) the photograph of a flattened FPCB. The red dashed boxes indicate the locations of the integrated circuit components. c) The system block diagram of IWCS. The whole system consists of devices, hardware system, and software system. Hardware system consisted of three‐electrode constant‐potential circuit, signal conditioning circuit, power supply module, Bluetooth, MCU, and two‐channel constant‐current sources. d) The home page (top) and the display page (down) of the smartphone app real‐time showing glucose monitor. e) The voltage output of as‐prepared FPCB at continuous and intermittent constant voltages of 0 and −100 mV, which could be applied as bias potential for sensing. f) The relations of the current signal on glucose electrodes using as‐prepared FPCB and commercial ECW. g) The linear relations of the currents and glucose concentration measured using as‐prepared FPCB compared to ECW. h) The current output of as‐prepared FPCB at continuous and intermittent constant current output at 0.5 mA for iontophoresis. i) The photograph of the whole IWCS (left), including RIMN sensor, FPCB, IMN device, and lithium‐ion polymer battery, communications with a smartphone app (right). A coin (marked with red circle) was placed next to the IWCS for visual size comparison. j) Photograph showing the simultaneous applications of the RIMN sensor and IMN devices on anesthetized rats. k) The dynamic glucose signals upon simultaneous applications of RIMN sensors and IMN devices on diabetic rat model. The detected current signal via RIMN was converted to glucose concentration, and the actual BG were measured via standard glucose test strips. The asterisk indicated the calibration point. The black arrow indicated the time point of insulin delivery by IMN device. The dash line indicated the boundary of normoglycemia (BG <200 mg dL^−1^). l) Statistical analysis showing the detection errors of RIMN sensor compared to the actual BG at corresponding time points. The asterisks indicated calibration points. The dash line indicated the clinical criterion of error <15%. m) The Clarke's error grid analysis showing the detection accuracy of RIMN sensor compared to the actual BG. The asterisks indicated calibration points. n) Biosafety Tests of MN Applications. Rat were treated with RIMN (0.5 mA) or MN for 5 min, totally four times with 10 min intervals. The images showing the skin surfaces 24 h after treatments of MN. Both RIMN and MN treatments did not cause obvious skin irritation. o) The skin tissues were sectioned and stained with hematoxylin eosin (H&E).

The FPCB module incorporated the required integrated circuit chips and peripheral electronics for reverse iontophoretic glucose extraction, electrochemical amperometric recording, signal processing, control and feedback, iontophoretic drug delivery, and Bluetooth wireless transmission circuitry, thus forming a fully integrated seamless and programmable IWCS circuit (**Figure** **5**a,b). Figure 5a illustrates the block diagram of the functional units of the IWCS, where the detail of each labeled component is discussed in Figures S25–S35, Supporting Information. The system block diagram of IWCS is as shown in Figure 5c. The whole system consisted of sensor and device, hardware system, and software system. The circuit consisted of a three‐electrodes system and two constant current sources. The constant source circuit on the left applied an electric field to extract subcutaneous interstitial fluid into MMN for sensing, while the constant current source circuit on the right achieved drug delivery by means of iontophoresis. The three‐electrode system enabled the output of stable bias potentials and a recording of amperometric signals that reflected glucose concentrations. The analog signals were sent to Microprogrammed Control Unit (MCU) and converted into digital domains through the digital‐to‐analog (DAC) port in microcontroller. The MCU outputted data through an on‐board wireless transceiver for real‐time observation. When hyperglycemia state was detected, a direct current signal was applied to trigger an iontophoretic release of insulin. The smartphone app displayed the app interface and real‐time monitoring of glucose data (bottom panel), conceptually demonstrated a fully integrated IWCS‐based hardware and software for diabetes management (Figure 5d and Figures S36 and S37, Supporting Information).

The typical output of as‐prepared FPCB at continuous and intermittent constant voltages of 0 and −100 mV are shown in Figure 5e with a stable voltage output that was suitable as bias potential for electrochemical sensing of IWCS (Figure S10, Supporting Information). The signal detection on glucose electrodes using as‐prepared FPCB (Figure 5f) was in consistency with that using a commercial electrochemical workstation (ECW), producing similar standard curves of signal‐glucose concentration (Figure 5g). The continuous and intermittent constant current output at 0.5 mA typically for iontophoresis was also demonstrated (Figure 5h). All these tests verified that the as‐prepared FPCB could provide satisfying electrical output and signal recording to support the sensing and delivery functionalities of IWCS. The FPCB were connected to both a RIMN sensor and IMN device to form the compact IWCS (with total dimensions of 28.5 × 42.2 × 7.7 mm and a mass of 20.6 g), with a 3.7 V lithium‐ion polymer battery as power supply. The IWCS could be flexibly wrapped on cylindrical objects or human arms with a compact size smaller than a smartphone (Figure 5i).

Simultaneous glucose monitoring and insulin administration using IWCS on diabetic rats in situ were further demonstrated, where commercial ECW instead of as‐prepared FPCB was employed since the FPCB size was designed for humans rather than rats. The RIMN sensor and IMN devices were both pressed against the rat's back to penetrate the skin by MMN (Figure 5j). Glucose sensing by RIMN sensor was performed every 20 min at the iontophoretic condition of 0.5 mA current and 300 s duration for each cycle, while standard glucose test strips were employed to measure the rat's BG (Red hollow point in Figure 5k) as positive control. The detected current signal via RIMN was converted to glucose concentration and calibrated using the first‐measured BG data point (at *t* = 20 min, indicated with a red star). The diabetic rats were administered by MN without iontophoresis for the initial 80 min and glucose level was observed to remain at the hyperglycemic state (Figure S39, Supporting Information). Subsequently, after application of IMN treatment for 60 min, the BG decreased significantly to normoglycemia within 1.5 h and then remained stable, suggesting the IMN accelerated insulin release to treat hypoglycemia (Figure 5k). The glucose fluctuation trend recorded by the RIMN was consistent with the standard BG measurements, where the accuracies were quantified and examined with Clarke's error grid analysis (Figure S40, Supporting Information). The errors of all the RIMN‐measured glucose signals were less than 40%, with an average error of 17.5 ± 13.9%, close to the clinical requirement of error <15% (Figure 5l). In the Clarke's error grid analysis (Figure 5m) 73.7% of diabetic group data were located in region A (error <20%). These results indicated the IWCS consisted of RIMN and IMN could simultaneously monitor glucose fluctuation in situ and provide controlled delivery to treat hyperglycemia, which were the essential functions for an intelligent system of diabetic management.

The biocompatibility and biosafety of the RIMN sensor were evaluated by histological examination of the induced skin irritation and inflammatory effects after MMN penetrations with reverse iontophoresis (Figure 5n). The RIMN sensor was pressed onto the rat's back, applied with a blank current (0 mA, as control), or a current of 0.5 mA for 5 min, with a 10 min interval per cycle for four cycles. The RIMN sensor was then removed, and the rats were returned to their cages for normal feeding. After 24 h, the local skin tissues treated with RIMN were excised, fixed, and stained with hematoxylin and eosin (H&E). Compared to normal skin tissue without RIMN treatment, neither the RIMN with 0.5 mA current nor MN with 0 mA current induced significant inflammatory cell infiltration in the treated tissue. This indicated that the application of RIMN did not cause significant skin irritation or inflammation (Figure 5o).

## 3 Conclusion

In this work, a fully integrated closed‐loop system based on MNs platform and wearable electronics that could simultaneously monitor and treat diabetes in situ was developed. The MMN served as an effective and mini‐invasive tool to directly access to the subcutaneous fluids, which enabled painless but accurate detection and regulation of glucose in vivo. When iontophoresis was coupled to the MMN, both sensing and delivery could significantly be improved by facilitating the mobilities of glucose and insulin across the MN interfaces. Electrical modulation and control of delivery were thus realized. By incorporation with FBCB that is responsible for signal recording/processing, feedback/triggering, signal transmission and wireless communication, this IWCS is highly intelligent and compact for diabetic treatments. Advances in this suite of system would provide new opportunities for improving the life qualities of diabetic patients, as well as holding great potential to be expanded to a variety of other chronic diseases. This would positively promote the emergence of a new generation of theranostic platforms for modern medicine.

## 4 Experimental Section

### Fabrication of MMN

Polydimethylsiloxane (PDMS, Sylgard 184, Dow Corning) and its curing agent were mixed by 10:1 and then stirred well. The uncured solution was placed in vacuum at 4.5 Pa for 30 min, aiming to remove bubbles. The PDMS solution was casted onto a SU‐8 master of MN array and then dried at 60 ℃ overnight, which formed a PDMS mold with inverted‐MN structure. The PDMS mold was then separated from the SU‐8 master mold and was ready to be applied as mold for fabricating MMN. For the typical preparation process of MMN, trimethylolpropane trimethacrylate (TRIM, Aladdin Reagents Co., Ltd, Shanghai), and triethylene glycol dimethacrylate (TEGDMA, Aladdin Reagents Co., Ltd, Shanghai) were used as a crosslinker for poly glycidyl methacrylate (PGMA, Aladdin Reagents Co., Ltd, Shanghai). Polyethylene glycol (PEG, 10 kDa, Sigma‐Aldrich) was used as porogen. First, 2 g PEG was dissolved in 10 ml 2‐methoxyethanol at 50 ℃ for 1 h as a porogen stock solution, ensuring the solution was transparent before use. Second, the monomer glycidyl methacrylate (1 ml; 73.3 mmol, 1 equiv. Aladdin Reagents Co., Ltd, Shanghai), TRIM (0.688 ml; 19.4 mmol, 0.26 equiv.), and TEGDMA (1.59 ml; 57.6 mmol, 0.79 equiv.) were uniformly mixed as a monomer stock solution. Third, Irgacure 184 (0.10 g, 1 wt% to the monomer, as photoinitiator, Sigma‐Aldrich) was added to the mixture of the monomer solution and porogen stock solution (1:1, v/v, totally 6.6 ml). After that, the mixture solutions were drop‐casted into the PDMS mold under a centrifugation at 4000 rpm for 10 min, ensuring the mixture solution into the inverted cavities of the PDMS mold. The microneedle patch was solidified in the PDMS mold by ultraviolet irradiation (INTELLI‐RAY 400, Uvitron, USA) at 365 nm for 20 min and peeled off from the PDMS mold. The solid microneedle patch was then immersed in 50% methanol solution for 24 h to remove the PEG porogen. In addition, other mixtures of the monomer and porogen stock solutions (7:3, 3:2, 2:3, v/v,) were prepared for fabricating MMN with 30%, 40%, and 60% porosities, respectively.

### Fabrication of Metal Microneedle Counter Electrodes

Laser microetching (INNO LASER) was employed to fabricate metal MN sheet from stainless steel substrate with ≈100 µm thickness. The metal MN was designed to possess ≈225 µm‐diameter at the base, ≈800 µm‐length, and ≈250 µm interval between adjacent MNs. The steel MN was then coated with ≈100 nm Au layer by Magnetron Sputtering (ZKDS VTC‐300, China).

### Fabrication of Glucose Sensing Electrode

Screen‐printed three electrode system on a plastic substrate (Ningbo Mxense Bio‐Tech Co., Ltd.) possessed two carbon electrodes as W.E. and C.E., and an Ag/AgCl electrode as R.E. The W.E. was sputtered with 30–50 nm‐thick Cr and 80 nm‐thick Au. The Cr layer served as a metal adhesion layer. Ferric ferrocyanide (also named Prussian Blue) was electrodeposited in situ on the W.E./Cr/Au electrode at constant voltage of 0.8 V for 480 s, in a fresh solution (100 mL) containing 2.5 mm FeCl3, 100 mm KCl (Aladdin Reagents Co., Ltd, Shanghai), 2.5 mm K_3_Fe(CN)6 (Aladdin Reagents Co., Ltd, Shanghai), and 100 mm HCl (Guanglianjin Chemical Industry Co., Ltd, Guangzhou). The electrochemical activities potential of the W.E./Cr/Au/PB electrode were evaluated via cyclic voltammetry. The cyclic voltammetry was performed with a scan rate of 50 mV s^−1^ at the potential range of −0.2 to 0.5 V, in KCl/HCl (0.1 m KCl and 0.01 m HCl) solution (in DI water). After washing and drying the electrode, 4 µl mixture solution containing glucose oxidase (50 mg mL^−1^ in PBS, Aladdin Reagents Co., Ltd, Shanghai)/ bovine serum albumin (80 mg mL^−1^ in PBS, Sigma‐Aldrich)/glutaraldehyde (2.5% in PBS, Aladdin Reagents Co., Ltd, Shanghai) was drop‐casted onto W.E./Cr/Au/PB electrode to fabricate the glucose sensing electrode. Afterwards the electrode was rinsed with PBS to remove un‐crosslinked enzymes on the surface and then dried in the ambient overnight.

### Assembly of RIMN Sensor

The MN CE, MMN, and 3D printed sensing chamber (by 3D printer, Formlabs Form 3) were assembled and glued together using a thin layer of light‐curing resin (white resin, Formlabs). The resin was cured under UV light irradiation (INTELLI‐RAY 400, Uvitron, USA) at 365 nm for 2 min to achieve seamless integration of the three components. The glucose sensing electrode was fixed inside chamber, in close contact with the bottom of MMN. The gap between electrode surface and MMN substrate were filled with 100 µL PBS solution. The chamber was sealed with a PDMS layer to prevent leakage of solution.

### Assembly of IMN Device

Similar with the RIMN sensor, the MN CE, MMN, and 3D printed delivery chamber were assembled and glued together using a thin layer of light‐curing resin. An Au‐coated electrode was placed on top of the MMN, and the gap between electrode surface and MMN substrate was filled with an insulin solution‐loaded melamine sponge. Afterwards the chamber was sealed with a PDMS layer.

### Design of FPCB

STM32F103C8T6 microcontroller (ARM 32‐bit) was the core of the authors' system which could be programmed on‐board through Serial Wire Debug (SWD) interface. This microcontroller possesses an integrated development environment, and was commonly used in autonomous systems with a low power and low‐cost requirement. The data (which was output by the sensor and adjusted to the appropriate value through the signal conditioning circuit) collected by the 12‐bit analog to a digital conversion module of a microcontroller was sent to the user terminal via Bluetooth through a serial port pass‐through. A constant potential detection circuit was used as the core circuit component, since the electrochemical glucose sensor relied on the amperometry method. The constant potential detection circuit consisted of voltage follower, control amplifier, feedback follower and spanning resistance amplifier. The control amplifier and the feedback follower formed a feedback structure. The potential of the reference electrode was controlled by the input voltage of the voltage follower. The working electrode was connected to the reverse input end of the transmissive amplifier, and the forward input end of the transmissive amplifier was grounded. Since the virtual ground effect was equivalent to the ground potential, the potential difference between the working electrode and the reference electrode was the input voltage of the voltage follower. The current output range of the sensor working electrode was at a nanoampere level, and the tran‐resistance amplifier was connected to a resistance of 1 MΩ. The signal resolution was conservatively estimated to be 1 nA. Two constant current sources were provided for either iontophoresis or reverse iontophoresis. The constant current source adopted a CMOS field effect tube of which the gate current was easy to control by voltage. The control voltage was output by the Digital‐to‐Analogue Conversion (DAC) of the microcontroller. By controlling the size and form of the voltage output, the size and form of the output of the constant current source could be controlled. A 3.7 V polymer lithium battery was employed as power supply. To ensure the circuit work properly 5, −5, 20, and 3.3 V power sources were needed, which were achieved by converting the 3.7 V from battery through boost circuit and step‐down circuit. The Bluetooth module used the low‐power Bluetooth chip CC2640R2F, which has a high transmission rate and very low power consumption that was suitable for wearable devices.

### Fabrication, Soldering, and Tests of FPCB

The fabricating process contained trepanning, exposure, developing, etching, stripping, lamination, and lead plating (by Shenzhen Star Extraordinary Technology Co. LTD, China). The through‐hole enabled two levels of lines to communicate with each other in the double‐sided FPCB. In specific, the high energy ultraviolet light beam emitted by a YAG ultraviolet laser was used to drill corresponding holes on the circuit board. Plasma was used to clean the hole residue and surface dirt after double plate drilling. Next, the copper sinking process by redox reaction was conducted, where a thin layer of copper was deposited into the hole and on the board surface, and the copper on the hole sidewall was connected to the copper on both sides of board surfaces. Then copper was further electrochemically plated into the hole and the board surface to increase the thickness of the metal. After that, pad pasting on the copper clad laminates was conducted for image transferring, followed by exposure and DES (Developing, Etching, Stripping). The designed PCB layout image was transferred onto a copper plate by dry film light induction. By the development process, the exposure parts were left for etching or electroplating of the resistance film. Extra copper was corroded, followed by an alkali treatment to lift‐off. Finally, lamination and lead plating were employed to solder the interfaces of the circuit board. An electric soldering iron was used to weld the circuit elements to their respective positions on the FPCB. Then, each functional module on the circuit board was tested and verified to ensure the normal implementation of the circuit function. A multimeter was used to test the output voltage of the power module. The performance of the constant‐current source was tested by connecting to the output port to test whether the currents could maintain constant. The performance of a signal processing circuit was tested for whether the output theoretical value fitted to the testing value.

### Design of Customized Mobile Application

To provide a user‐friendly interface for data recording and display by IWCS, a mobile application was designed to support the IWCS. Application received and resolved the stream of data that transmitted in real‐time from the IWCS. While the IWCS hardware did not filter the analog signal, the app could assist in filtering the data before calculating the BG value to eliminate noise interference. The Butterworth low‐pass filter with the cut‐off frequency of 1 Hz was adopted to filter the BG signal, since the BG signal is a slowly changing signal, close to a direct‐current signal. The output data of the digital filter was converted to BG value and displayed on the interface in real‐time, and the history of previous BG values could also be viewed by sliding the display interface. The above interface was implemented on the Android platform, but similar applications can be developed on other popular operating platforms such as iOS.

### Integration of IWCS

RIMN sensor (connected to three‐electrode interface and constant‐current source interface), IMN device (connected to constant‐current source interface), and Li‐ion polymer batteries (power interface) were soldered on the corresponding interfaces of FPCB to integrate the IWCS.

### Theoretical Calculation Via COMSOL Multiphysics 5.5 Simulation

The models were constructed by COMSOL Multiphysics 5.5 using the AC/DC module and Chemical Species Transport module. The process of both glucose extraction and cargo delivery were simulated with a 2D model, where the components and geometries mimicked the cross section of the actual setup. The skin tissue was modeled as three layers (stratum corneum, epidermis, and dermis). The MMN inserted into the skin, with a MN CE placed next to it. Constant electrical currents were supplied for iontophoresis or reverse iontophoresis. For glucose extraction, the glucose concentration was set as Cg0 in dermis domain, and the related boundary was set as influx boundary in same concentration to simulate the supplement of glucose. Under an electrical field, glucose would diffuse through the micro‐channels of MMN into the backside sensing chamber. After extraction, the average glucose concentration in the sensing chamber was calculated to evaluate the extraction efficiency. The extracted glucose concentration was normalized by comparing to the initial glucose concentration in the interstitial space. The insulin delivery model was similar with the extraction model. The top boundary of the chamber was set with an influx of insulin concentration Ci0. The boundaries under the skin were set open assuming the insulin could diffuse further to an unlimited space of body system. After releasing, the average insulin concentration in the interstitial space was calculated to evaluate the delivery efficiency. The released insulin amount was normalized by comparing to the initial insulin amount in the IMN device chamber. The related physical parameters for simulation include: 1) the molecular (glucose and insulin) diffusivities and electrical conductivities of the PBS solution, MMN materials, stratum corneum, and viable epidermis and dermis; 2) the porosity of MMN; 3) initial concentrations of glucose in the interstitial space and initial concentrations of insulin in the delivery chamber; and 4) the electrical charge of glucose and insulin. The detailed physic setting of glucose extraction and cargo delivery, related parameters, and the governing equations can be found in the Supporting Information.

An AC/DC module was employed to compute the steady electric filed distribution, which was calculated by electric currents interface, following the controlling equations:

(2) ∇·J=0

(3) J=σE

(4) E=−∇V

where *V* refers to potential, *E* is the intensity of electric field, *J* refers to current density, *σ* is the material conductivity, and *∇* refers to Hamiltonian. The above equations resulted in a Laplace equation for the calculation of electric potential and electric field:

(5) ∇(σ·∇V)=0

On the top boundary of RIMN chamber, a boundary current terminal was used to simulate the constant current source:

(6) $$\int_{\partial \Omega }J\cdot ndS=I_{0}\;$$

where *I*_0_ refers to constant current, *n* is the normal vector of boundary unit, and *Ω* refers to the whole boundary. This equation suggests that the normal integral of the current density of all elements at the terminal boundary was equal to the given current magnitude. The boundary of MN CE was set ground connection and the other boundary was insulated.

Next, time‐dependent study was used to simulate the dynamic iontophoresis process via Chemical Species Transport module, invoking the electric filed distribution. The govern equation was as below:

(7) $$\frac{\partial c}{\partial t}+\nabla \cdot \;J_{\mathrm{tds}}=\;0$$

(8) $$J_{\mathrm{tds}}=-D_{e}\nabla_{C}-zu_{\mathrm{me}}Fc\nabla V$$

where *J*_tds_ refers to diffusion flux vector, *c* is the concentration, *z* is the charge number, F refers to Faraday constant, *V* is the electric potential, *D*_e_ is the effective diffusion coefficient, *u*_me_ is the effective mobility, the relationship of *D*_e_ and *u*_me_ can be described as the Nernst–Einstein equation:

(9) $$u_{\mathrm{me}}=\frac{D_{e}}{RT}\;$$

where R is Moore gas constant, *T* is temperature.

In the authors' model, the MN array domain was set as porous material, which would impede the fluid flow, thus impaired matter diffusion. The equation is based on Millington Quirk model:

(10) $$D_{e}\;=\varepsilon_{p}^{4/3}\;\cdot D_{F}$$

where ϵ*_p_* refers to porosity, *D*_F_ refers to the fluid diffusion coefficient.

### Characterization of MMN Morphology

After the MMN was fabricated, the morphologies were characterized by scanning electron microscope (SEM, SUPRA 60, Zeiss, or Phenom Pro Desktop SEM, Thermal Fisher Scientific), or by optical microscope (Axio Scope A1, Zeiss). Samples were deposited a thin layer of Au (<10 nm) for SEM examination, using an SBC‐12 Sputter Coater (Beijing Zhongjingkeyi Technology Co., Ltd).

### Mechanical Characterization of MMN

For fracture test by compression force, a metal platform was pushed to the MMN tip at the rate of 4 mm min^−1^. Displacements and forces on the metal platform were simultaneously recorded by a force gauge (INSTRON, 5943, USA). The maximum force before the sharp drop of force was identified as the breaking force, and the force at the tangent point of the displacement curve was identified as the yielding force.

### Characterization of Molecular Diffusion Rates through MMN

Based on the dye specific light absorption, the optical absorption–concentration curves of insulin‐FITC (*M*_w_: 6122.88, 200 µL, 5 mg mL^−1^, Aladdin Reagents Co., Ltd, Shanghai) and methylene blue (*M*_w_: 319.8, glucose substitute, 200 µL, 1 mg mL^−1^, Aladdin Reagents Co., Ltd, Shanghai) were established, respectively. The insulin‐FITC and methylene blue centration were quantified by the optical absorption density (O.D.) at 495 and 664 nm, respectively. Absorption–concentration analyses were performed via an Infinite M200 Pro Multi Detection Microplate Reader (Tecan, Switzerland). The tips of MMN with different porosities of 30%, 40%, 50%, and 60% penetrated a parafilm (≈127 um thickness; mimicked the stratum corneum) covered on a chamber containing PBS solution. A drop of PBS solution containing methylene blue or insulin‐FITC was dropped on the MMN bottom substrate. After a 1 h free diffusion, the optical absorption value of the dye in chamber was measured, and was converted to dye concentration via the standard concentration curve. The release amounts of dye were normalized by comparing to the total amounts in the drops applied onto MMN.

### Characterization of MMN Penetration into Tissue

The tip of MMN was pre‐stained with red fluorescent dye by soaking the MMN with Rhodamine B (1 mg mL^−1^, Aladdin Reagents Co., Ltd, Shanghai) for 5 min. The MMN were pressed against a piece of pigskin for 5 min and then withdrawn. The tissue was then imaged with fluorescence microscopy (Mshot MF‐41). The pigskin after MMN‐treatment was further cut into 2‐cm sections corresponding to the location of the MMN, fixed on slides, and the cross‐section was imaged with fluorescence microscopy.

### Electrochemical Detection of Glucose In Vitro

The electrochemical activities potential of the glucose electrode were evaluated via a standard three‐electrode electrochemical workstation (CH Instrument, 760E) at room temperature. Cyclic voltammetry scanning in KCl/HCl solution (0.1 m KCl and 0.01 m HCl in DI water were performed at the potential range of −0.2 to 0.5 V. The sensing of glucose (in PBS solution) was performed at the bias potential of 0 V due to the presence of Prussian Blue. Glucose concentration was stepwise increased by 0.2 mm up to 1.0 mm. The amperometric signal was recorded, which required >2 min for signal stabilization.

### Glucose Extraction and Sensing Via RIMN In Vitro

A chamber (10 ml) containing a sponge (70% porosity) was used as artificial skin tissue, and a water‐impermeable parafilm was placed on top to mimic the stratum corneum. A PBS solution containing 10 mm glucose was filled in the sponge. MMN penetrated the parafilm and the MN tips were immersed into the glucose solution. The glucose was extracted into the RIMN sensor at different iontophoretic currents (0, 0.2, 0.5 mA) and different iontophoretic durations (2, 5 and 10 min). Then the solution in the sensor chamber was withdrawn at corresponding time points for measuring extracted glucose concentration using the glucose assay kit (Beijing Boxbio Science & Technology Co., Ltd., Beijing, China). The optical absorption–concentration standard curve was established for calibrating the glucose concentration.

For electrochemical sensing, a series of glucose concentrations (2 to 10 mm) of glucose solutions were filled into the sponge in chamber. The MN CE was also inserted into the sponge. The glucose was extracted by reverse iontophoresis at different iontophoretic currents (0 and 0.5 mA) for 5 min. Subsequently, the glucose concentration was electrochemically detected via the glucose electrode of RIMN sensor at 0 V.

### In Vitro Release Studies

A chamber (3 ml) containing an agar (1 wt%, 1 ml) was used as artificial skin tissue and a parafilm was placed on top to mimic the stratum corneum. Insulin solution (4 mg mL^−1^) was loaded into the IMN device, and was released into the agar in a chamber through MMN. The insulin was released by IMN either at 0 or 0.5 mA. At corresponding time points (30, 60, 90, 120, and 180 min), the agar was withdrawn and smashed via ultra‐sonication (Qsonica, Q700), in order to extract the insulin for determination of concentration. The amount of insulin was examined using a protein assay (Coomassie Plus protein assay, ThermoFisher), referenced to an established standard optical absorption–concentration curve.

### The Performance Tests of FPCB

A stable voltage between working electrode and reference electrode was provided by the three‐electrode constant potential circuit. The voltage amplitude and mode were completely controlled by the MCU. The Digital‐to‐Analog converter (DAC) of the MCU outputs two voltage modes including 100 mV DC signal and rectangular waves (100 mV amplitude, 50% duty radio, and 60 s period) for testing the constant‐current sources of FPCB. Next, planar glucose electrodes were connected to the electrochemical workstation (ECW) and FPCB, respectively, and the sensing of different glucose concentrations were recorded. The relations of glucose concentrations and recorded amperometric signals via ECW and FPCB were linearly fitted. The relations were normalized for comparison to verify whether the FPCB could satisfyingly record glucose signals. Third, to verify the performance of constant‐current sources, a 100 Ohms precision resistor was connected to the output of a constant‐current source. The DAC of the MCU outputs 500 mV DC signal and rectangular waves (50% duty radio and 60 s period). The voltage signals at both ends of the resistance were collected by the DAQ, and the output current values were calculated by the Ohm's theorem.

### Experimental Animals

Male Sprague‐Dawley (SD) rats weighing 200–250 g (Animal Center, Sun Yat‐sen University, Guangzhou, China) were used for experiments. Rats were housed in a climate‐controlled room under a 12 h/12 h light/dark cycle and with food and water ad libitum. All animal experiment in this work were reviewed, approved, and supervised by the Institutional Animal Care and Use Committee at the Sun Yat‐sen University. For all experiment procedures, the animals were deeply anesthetized with 2% isoflurane.

Streptozotocin (STZ, Sigma‐Aldrich) at a dose of 60 mg kg^−1^ was used to induce type 1 diabetes rats. The treated rats were fasted and allowed to drink freely for one night. The BG of the rats were monitored continuously for 7 days, and the rats with stable BG higher than 300 mg dL^−1^ were selected for experiments. Blood samples were collected from the tail vein of the rats to measure the BG by a standard Roche blood glucose meter (Accu‐Chek Performa). The glucose levels detected via RIMN were compared to the actual for quantifying the detection accuracies and were examined with Clarke's error grid analysis.

### Application of RIMN Sensor on Rat Models

The rats were continuously anesthetized with 2% isoflurane gas and the hairs on the rats’ dorsal surfaces were removed by hair removal cream (VEET depilatory cream, Reckitt Benckiser) the day before experiment. The RIMN sensor was pressed against the rat's back and fixed on an iron platform with copper coils, and the whole test process remained stable.

For healthy rats, glucose fluctuations were induced at different time points (at 30 and 50 min) by an intraperitoneal injection of 5 ml glucose (5%) solution. Glucose levels were detected via RIMN sensor. At each measurement, glucose was extracted by RIMN sensor at 0.5 mA for 5 min (using a multichannel source meter, Keithley 2636B). At a bias potential of 0 V, the extracted glucose concentration was electrochemically detected using an ECW to record the amperometric signal, which required >2 min for signal stabilization. After the RIMN sensor detection, blood was drawn from the tail tip of the rat at different time points (20, 30, 40, 50, 65, 80, 90, and 105 min) for BG determination using a Roche blood glucose meter.

The diabetic rats were subcutaneously injected with 5 UI insulin at the time *t* = 20 min. At each measurement, glucose extraction via RIMN sensor was performed at 0.5 mA (using a multichannel source meter, Keithley 2636B) for 5 min. The extracted glucose concentration was electrochemically detected by recording the amperometric signals at the bias potential of 0 V. Glucose was detected via RIMN sensor at different time points (0, 20, 40, 40, 60, 80, 100, 120, 140, 160, and 180 min). Meanwhile, blood was drawn from the tail tip of rats for BG measurement using a Roche blood glucose meter.

### In Vivo Transdermal Delivery of Insulin

The dorsal hair of all rats was shaved off the day before drug administration. The rats were divided into 5 groups: a) healthy rats without any treatments; b) diabetic rats without any treatments; c) diabetic rats subcutaneously injected with 5 IU insulin; d) diabetic rats applied with IMN device for 3 h with 0.5 mA iontophoresis; and e) diabetic rats applied with an MN device for 3 h without iontophoresis. BG of the rats were measured every hour by a Roche blood glucose meter. The normoglycemia time was analyzed by determining the time window with BG <200 mg dL^−1^. During drug administration, 200 µL tail vein blood was collected at *t* = 2 h to determine the concentration of serum insulin in rats. Serum insulin concentrations were measured using an insulin ELISA kit (Meimian Industrial Co., Ltd, China). For statistical analysis, the significance of the difference was calculated by one‐way ANOVA.

### Simultaneous Application of RIMN Sensor and IMN Device on Diabetic Rat Model

Diabetic rats were anesthetized with 2% isoflurane and the hairs on the rat's dorsum were shaved. The RIMN sensor and IMN device were both pressed against the diabetic rats’ back and these were secured to them with copper coils on an iron platform, which remained stable during the experiments. The diabetic rats were administered by IMN device without iontophoresis for the initial 80 min. Subsequently, iontophoretic current of 0.5 mA was applied to the IMN for 60 min to facilitate insulin delivery. At each measurement, glucose extraction via RIMN sensor was performed at 0.5 mA for 5 min. Glucose was detected via RIMN sensor every 20 min, the amperometric signal was recorded at the bias potential of 0 V. Meanwhile, blood was drawn from the tail tip of the rats every 20 min for BG measurement using a Roche blood glucose meter.

### Biocompatibility and Biosafety Tests of IWCS Applications

The skin irritation caused by RIMN sensor application was tested. The RIMN sensor was pressed onto the rat's back, applied with a blank current of 0 mA or a direct current of 0.5 mA for 5 min (using a multichannel source meter, Keithley 2636B), with a 10 min interval per cycle for four cycles. The RIMN sensor was then removed, and the rats were put back into their cages for normal feeding. After 24 h, the local skin tissues treated with RIMN (0.5 and 0 mA) were excised, fixed, and then stained with hematoxylin and eosin (H&E). The tissue profiles were observed with optical microscope (M‐shot).

## Conflict of Interest

The authors declare no conflict of interest.

## Supporting information

Supporting Information

## Data Availability

The data that support the findings of this study are available from the corresponding author upon reasonable request.
