# Supplementary material for: A Fully Integrated Closed‐Loop System Based on Mesoporous Microneedles‐Iontophoresis for Diabetes Treatment
Source: Adv Sci (Weinh). 2021 Jun 3;8(16):2100827. doi: 10.1002/advs.202100827 (PMC8373098; doi:10.1002/advs.202100827)
Supplement: Supplementary file 1 — Supporting Information [file ADVS-8-2100827-s001.pdf]

WILEY-VCH

© 2021 Wiley-VCH GmbH

ADVANCED SCIENCE
Open Access

Supporting Information

for *Adv. Sci.*, DOI: 10.1002/advs.202000827

A Fully Integrated Closed-loop System based on Mesoporous Microneedles-Iontophoresis for Diabetes Treatment

*Xiangling Li+, Xinshuo Huang+, Jingshan Mo, Hao Wang, Qiqi Huang, Cheng Yang, Tao Zhang , Hui-Jiuan Chen, Tian Hang, Fanmao Liu, Lelun Jiang, Qianni Wu, Hongbo Li, Ning Hu and Xi Xie**

Supporting Information

A Fully Integrated Closed-loop System based on Mesoporous Microneedles-Iontophoresis for Diabetes Treatment

*Xiangling Li+, Xinshuo Huang+, Jingshan Mo, Hao Wang, Qiqi Huang, Cheng Yang, Tao Zhang , Hui-Jiuan Chen, Tian Hang, Fanmao Liu, Lelun Jiang, Qianni Wu, Hongbo Li, Ning Hu and Xi Xie**

X. Li, X. Huang, J. Mo, H. Wang, Q. Huang, C. Yang, T. Zhang, Prof. H. Chen, T. Hang, F. Liu, Q. Wu, H.Li, Prof.N.Hu and Prof. X.Xie
The First Affiliated Hospital of Sun Yat-Sen University; State Key Laboratory of Optoelectronic Materials and Technologies, School of Electronics and Information Technology; Guangdong Province Key Laboratory of Display Material and Technology, Sun Yat-Sen University, Guangzhou, China
E-mail: xiexi27@mail.sysu.edu.cn (X.Xie)
X. Li, T. Zhang and Prof. L. Jiang
School of Biomedical Engineering, Sun Yat-SenUniversity, Guangzhou, China
Q. Wu
Zhongshan Ophthalmic Center, Sun Yat-Sen University, Guangzhou, China
+These authors contributed equally to this work.

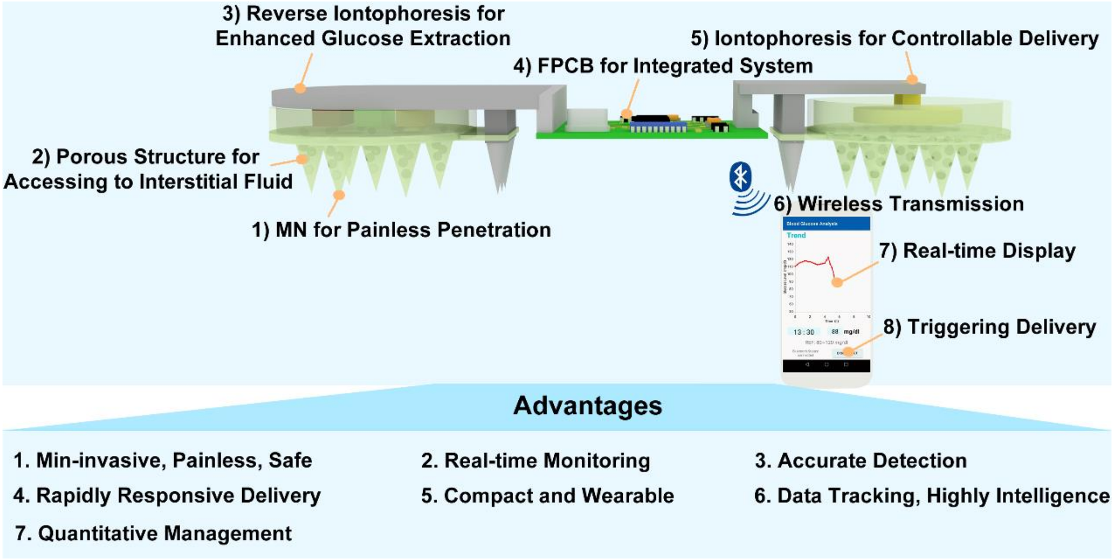

Figure S1. Summaries of the functions of each component and the advantages of IWCS. The functional advantages of IWCS include: 1) MN structure provided painless and safe penetration of skin since the avoidance of piercing nerves or blood vessels in the interstitial space. 2) Real-time, continuous and in-situ monitor of the dynamic fluctuation of glucose levels. This would be favorable for diagnosis of diabetes progress and the evaluations of medical treatments. 3) More accurate detection of glucose fluctuation compared to non-invasive method since the MN was directly access to the interstitial space. Although the glucose in interstitial fluid possessed ~5 min lag time, this could be ameliorated by algorithm calibrations. 4) Rapid and automatically controlled insulin delivery for regulation of hyperglycemia. The continuous diffusion of through MMN form a basal delivery to control the basic glucose level, while iontophoresis was coupled to MMN to facilitate a bolus delivery to minimize glucose fluctuation. 5) The IWCS was compactly designed, with satisfying miniaturization and flexibility to be worn on arms of human adults. 6) The system was intelligent and automatic. By Bluetooth communication with the smartphone, the glucose sensing data could be stored, displayed and tracked. The smartphone could also analyze data and trigger delivery. 7) By analyzing and co-relating the continuous data of glucose fluctuations with daily activities, diabetes managements could be quantitatively performed.

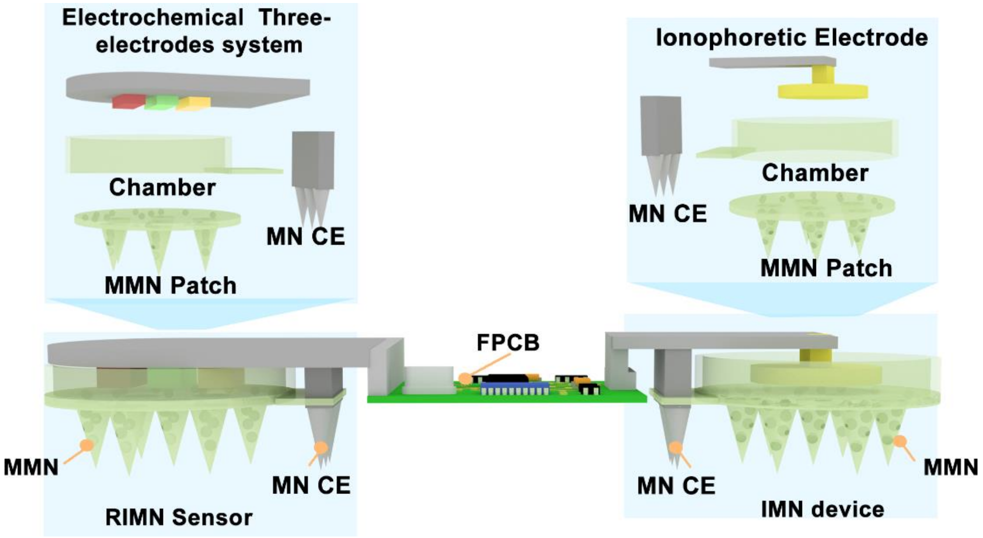

Figure S2. The detailed information of the assembly of IWCS and the key components (RIMN sensor and IMN device). The MN CE, MMN and 3D printed sensing chamber were assembled to form the RIMN sensor. The glucose sensing electrode was fixed inside chamber, in close contact with the bottom of MMN. Similar with the RIMN sensor, the MN CE, MMN and 3D printed delivery chamber were assembled to form the IMN device. An Au-coated electrode was placed on top of the MMN. RIMN sensor (connected to three-electrode interface and constant-current source interface), IMN device (connected to constant-current source interface) were soldered on the corresponding interfaces of FPCB to integrate the IWCS.

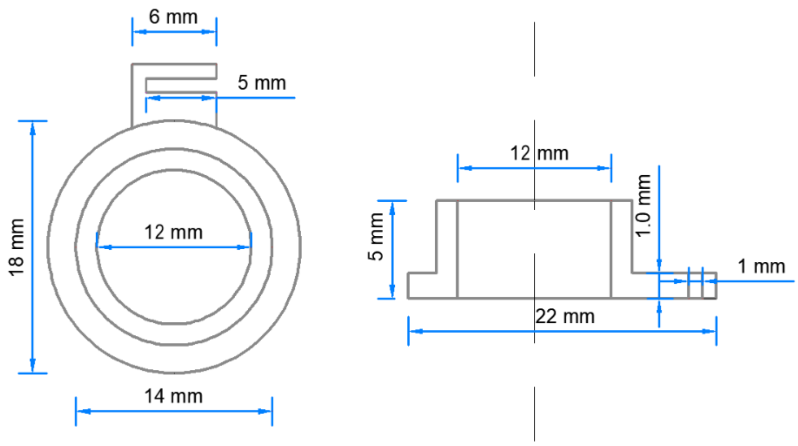

Figure S3. The CAD diagram (left: top view; right: side view) showing the actual design dimensions of the 3D-printed plastic chamber of both NM sensor and MN device.

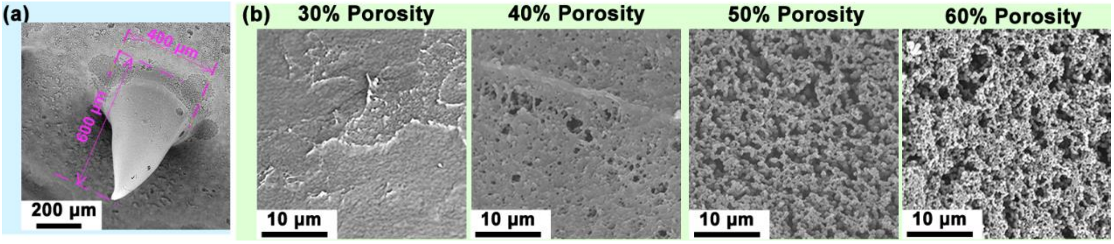

Figure S4. (a) The SEM image of a typical MMN tip, where the base-diameter and length were measured. (b) SEM images showing the zoom-in surface morphologies of MMN with porosity ranging from 30% to 60%.

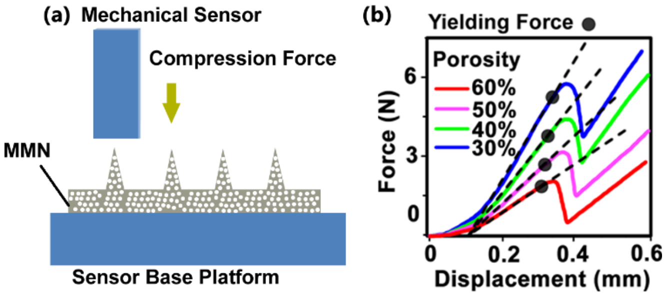

Figure S5. (a) Schematic illustration of the setup for measuring mechanical fracture forces. (b) Analysis of the yielding force (the tangent) in the stress-strain behavior test of a series gradients porosity of MMN (30-60%) by dynamometer. The tangent lines were labeled with dash lines, and the tangent points indicated the corresponding yielding force.

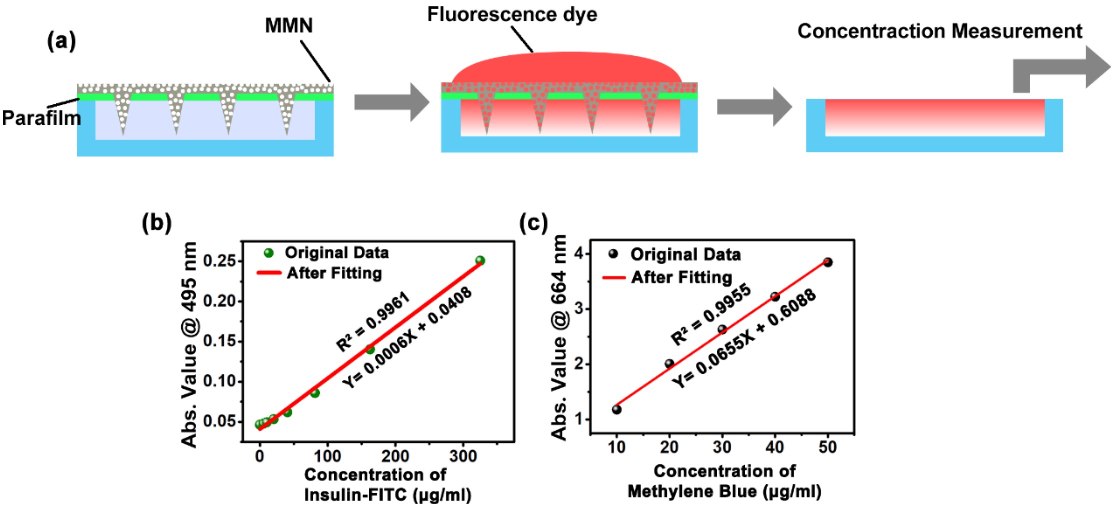

Figure S6. (a) Schematic showing the in vitro experiment studying the molecular diffusion through MMN. The MMN penetrated a parafilm, with the MN tips immersed into the PBS solution. Fluorescent reagents were applied on the bottom of MN substrate, and diffuse into the PBS solution through the MMN tips. The amount of the fluorescent reagents diffused into the PBS solution was quantified. (b) The relation of the optical absorbance (measured at 495 nm) with insulin-FITC concentrations. (c) The relation of the optical absorbance (measured at

664 nm) with methylene blue concentrations. The relation was linearly fitted as a standard curve.

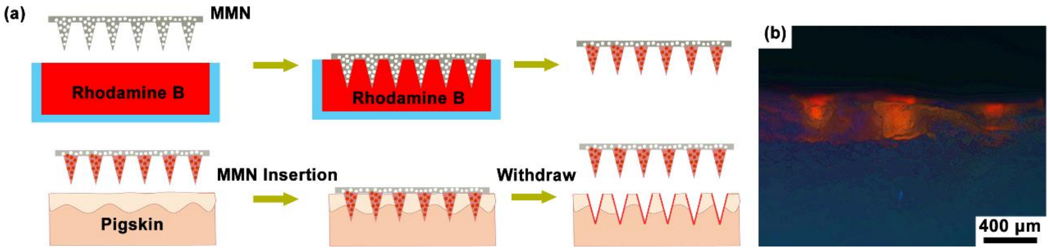

Figure S7. (a) Illustration of the experiment on MMN insertion into skin. The MMN was stained with red fluorescent dye. The MMN were then inserted into skin and withdrawn after 5 min. The fluorescence dye deposition in skin was observed with fluorescence microscopy. (b) Cross-section view of fluorescence images showing the MMN-mediated deposition of fluorescent dye in skin. The skin tissue was sliced and the cross-section profile was imaged with fluorescence microscopy. The penetration depth was around 400 μm.

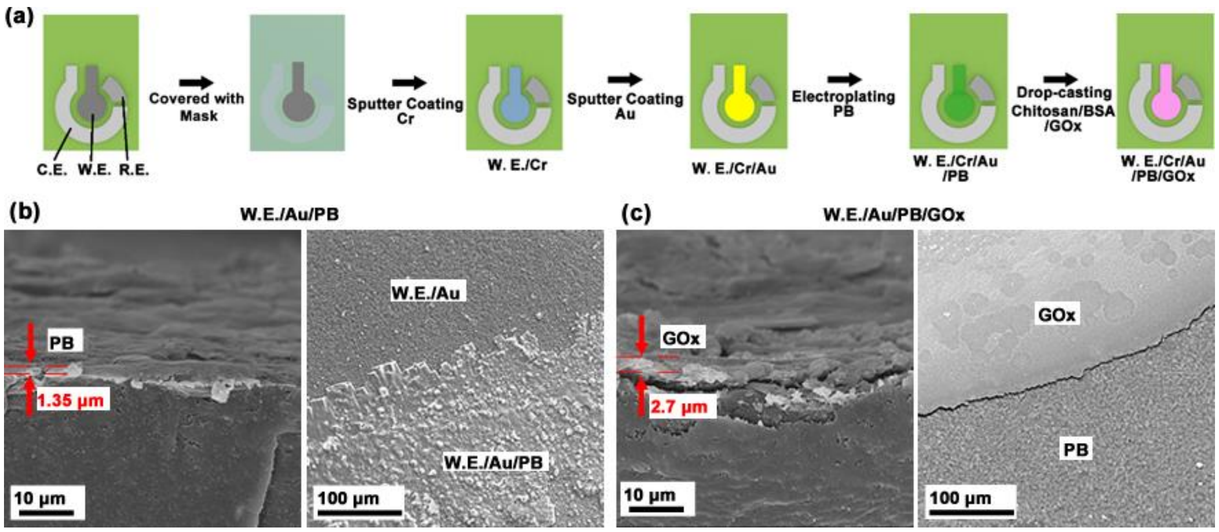

Figure S8. (a) Schematic showing the full process of fabricating glucose electrode. A screen-printed carbon electrode was covered with a metal mask, and then sputtered with a 50 nm-thick Cr layer and an 80 nm-thick Au layer. The Cr served as adhesion layer between Au layer and carbon electrode. Prussian blue (PB) was then electrodeposited in situ on the Au electrode surface, which served as a redox-active material to provide better selectivity and sensitivity. The electrode was then coating with a mixture of glucose oxidase, bovine serum albumin and glutaraldehyde by drop-casting. The bovine serum albumin and glutaraldehyde were used to immobilize the enzyme on electrode surface. (b) The Cross-sectional and SEM image of PB on the W.E,/Au/PB and (c) GOx on the W.E,/Au/PB/GOx. The thickness of each layer in dried states were measured.

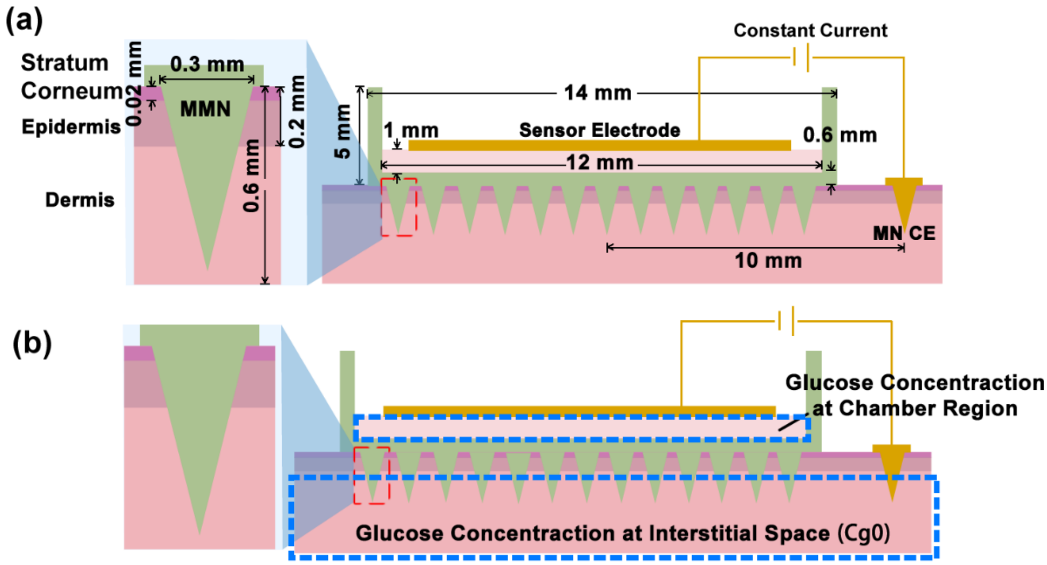

Figure S9. (a) Schematic of COMSOL simulation to calculate the electrical field and percutaneous potential on the RIMN sensor and related detailed dimension parameters in the simulation. The array included 13 MMNs in a cross-section, with 0.8 mm-interval between adjacent MMNs. The inner diameter and outer diameter of the chamber was 12 mm and 14 mm, respectively. The thickness of the MMN substrate heigh was 0.6 mm. The thicknesses of different skin layers were 0.02 mm (stratum corneum), 0.2 mm (viable epidermis) and 2 mm (dermis). The separation between electrode and MMN substrate was 1 mm. Constant current was applied between the electrode and the MN CE. Related physical parameters for simulation includes: 1) the molecular (glucose and insulin) diffusivities and electrical conductivities of the PBS solution, MMN materials, stratum corneum, viable epidermis and dermis; 2) the porosity of MMN; 3) initial concentrations of glucose in interstitial space and initial concentrations of insulin in delivery chamber; 4) Electrical charge of glucose and insulin. (b) The average glucose concentrations in the area marked with blue dash lines were calculated, respectively. The glucose concentration was set as C_{g0} in dermis domain, and the related boundary was set as influx boundary in same concentration to simulate the supplement of glucose. Under electrical field, glucose would diffuse through the micro-channels of MMN into the backside sensing chamber. After extraction, the average glucose concentration in the sensing chamber was calculated to evaluate the extraction efficiency. The average glucose concentration in the sensing chamber was normalized to the average glucose concentration in the interstitial space.

Supplementary Table. 1

Symbol	Value	Definition
Con_e	5e-4 [S/m]	**Conductivity of stratum corneum.**[1]
Con_s	0.2 [S/m]	**Conductivity of dermis and viable epidermis.** [1]
Con_w	1.6 [S/m]	**Conductivity of solution.** [2]
Dg	5e-10 [m^2/s]	**Diffusion coefficient of glucose in water.** The diffusion coefficient of small molecules in water is generally in the range of $1e^{-9}$-$1e^{-11}$[m^2/s].[2] The results using different diffusion coefficients (ranging from $3e^{-10}$ to $9e^{-10}$[m^2/s])were also investigated below.
Di		**Diffusion coefficient of insulin in water.** The diffusion coefficient of glucose in water is generally in the range of $3e^{-10}$ to $9e^{-10}$[m^2/s]. Considering that the diameter of an insulin molecule is roughly 3-folds larger than a glucose molecule, the Diffusion coefficient of insulin is assumed to be 3-folds lower than the glucose. The results using different diffusion coefficients (ranging from $1e^{-10}$ to $2.5e^{-10}$[m^2/s]) were also investigated below.
Dde	0.5	**Relative diffusion coefficient of molecules in dermis compared to that in water.**[3,4]
Dve	0.025	**Relative diffusion coefficient of molecules in viable epidermis compared to that in water.**[3,4]
Dsc	0.00025	**Relative diffusion coefficient of stratum corneum compared to that in water.**[3,4]
Ep	0.5	**Porosity of MMN.** Other porosities (0.3, 0.4, 0.6) were also investigated.
Zg	0	**Electric charge of glucose.** Glucose molecule is uncharged in water. During iontophoresis, Na^+ and Cl^- underneath skin migrate toward the electrodes by the

		applied potentials. The iontophoresis of glucose is achieved by convective transport of glucose along with the ions. The electric charge of Na^+ is +1. The glucose moved to the cathode along with the Na^+, with a convective ratio assumed to be 1. The results using different convective ratio (ranging from 1 to 1/16) were also investigated below.
Zi	-2	**The net negative charge of insulin in PBS solution (pH=7.4) is about -2.**[5,6]
Cg0	10 mM	**Initial concentration of glucose in the interstitial space.**
Ci0	4 mg/ml	**Initial concentration of insulin in the delivery chamber.**
Ii	0.5 [mA]	**Iontophoresis current for insulin delivery.** The results using different iontophoresis currents were also investigated.
Iri	0.5 [mA]	**Reverse iontophoresis current for glucose extraction.** The results using different iontophoresis currents were also investigated.

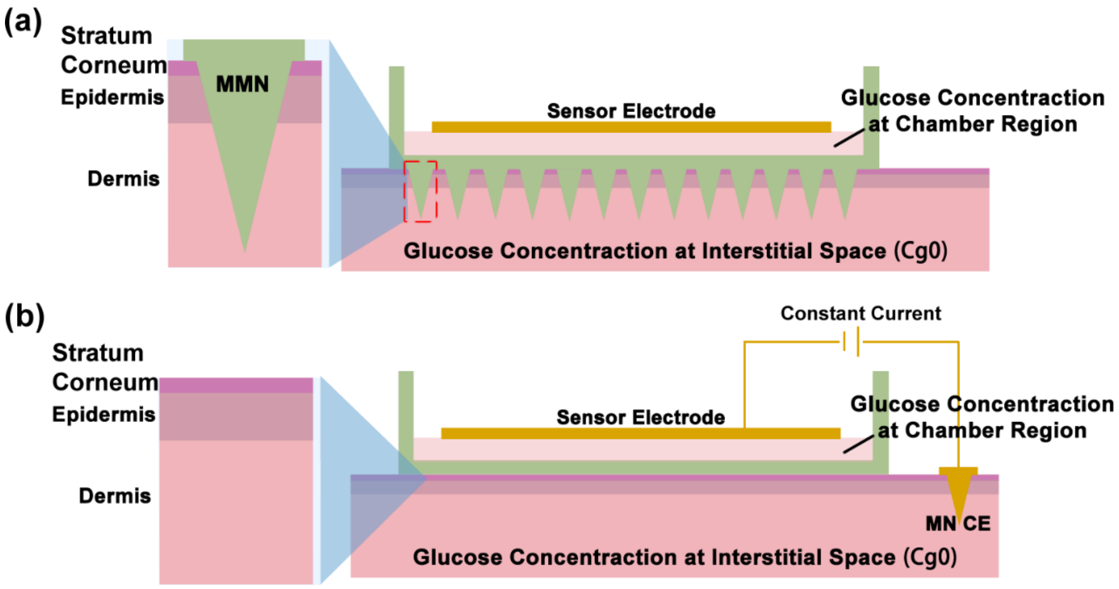

Figure S10. (a) COMSOL 2D model of glucose extraction by MN sensor without iontophoresis. Glucose diffused from the interstitial space to the sensing chamber through the MMN. (b) COMSOL 2D model of glucose extraction by reverse iontophoresis sensor without

MMN. Constant current was applied between the electrode and the MN CE. Glucose was extracted across skin layer into the sensing chamber. Na^+ and Cl^- underneath skin migrate toward the electrodes by the applied potentials. The iontophoresis of glucose is achieved by convective transport of glucose along with the ions.

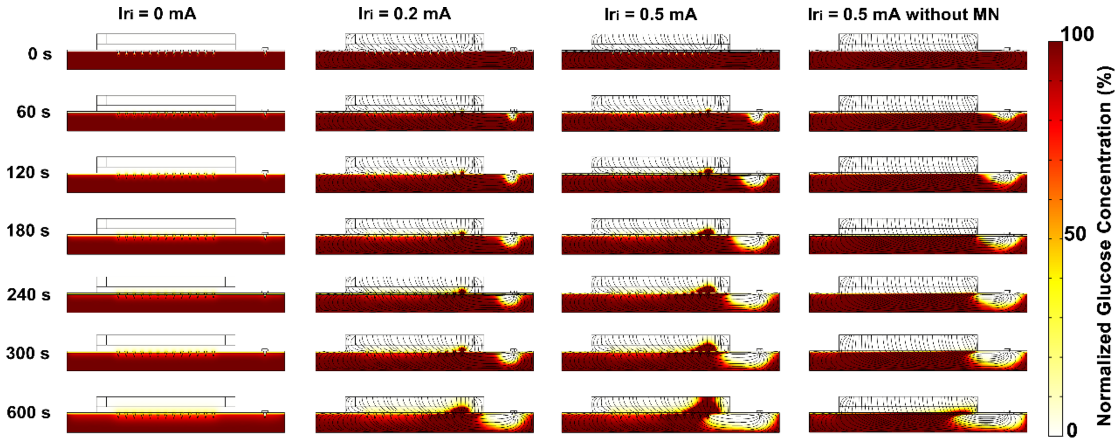

Figure S11. The electrical field distribution (indicated with black curves) and glucose concentration profile (indicated with brown color) at different time points (0-600 s) during reverse iontophoresis with different current conditions (0-0.5 mA) via RIMN sensor.

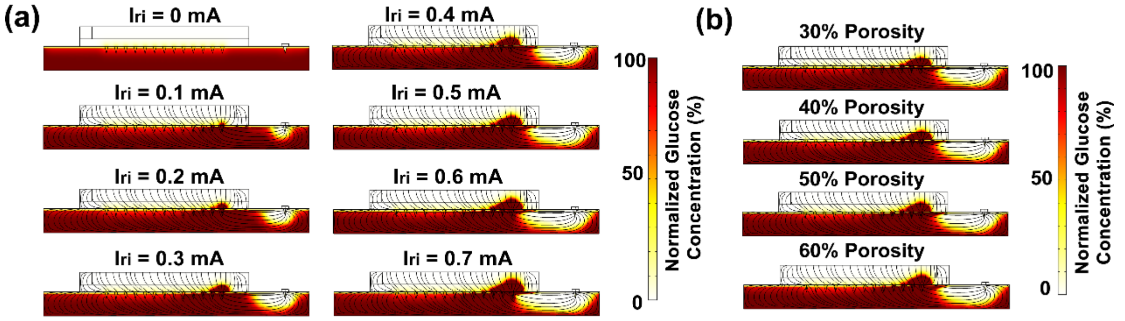

Figure S12. (a) The electrical field distribution (indicated with black curves) and glucose concentration profile (indicated with brown color) at t=300 s after reverse iontophoresis at different current conditions (0-0.7 mA). (b) The electrical field distribution (indicated with black curves) and glucose concentration profile (indicated with brown color) at t=300 s using RIMN with different MMN porosities (30% - 60%).

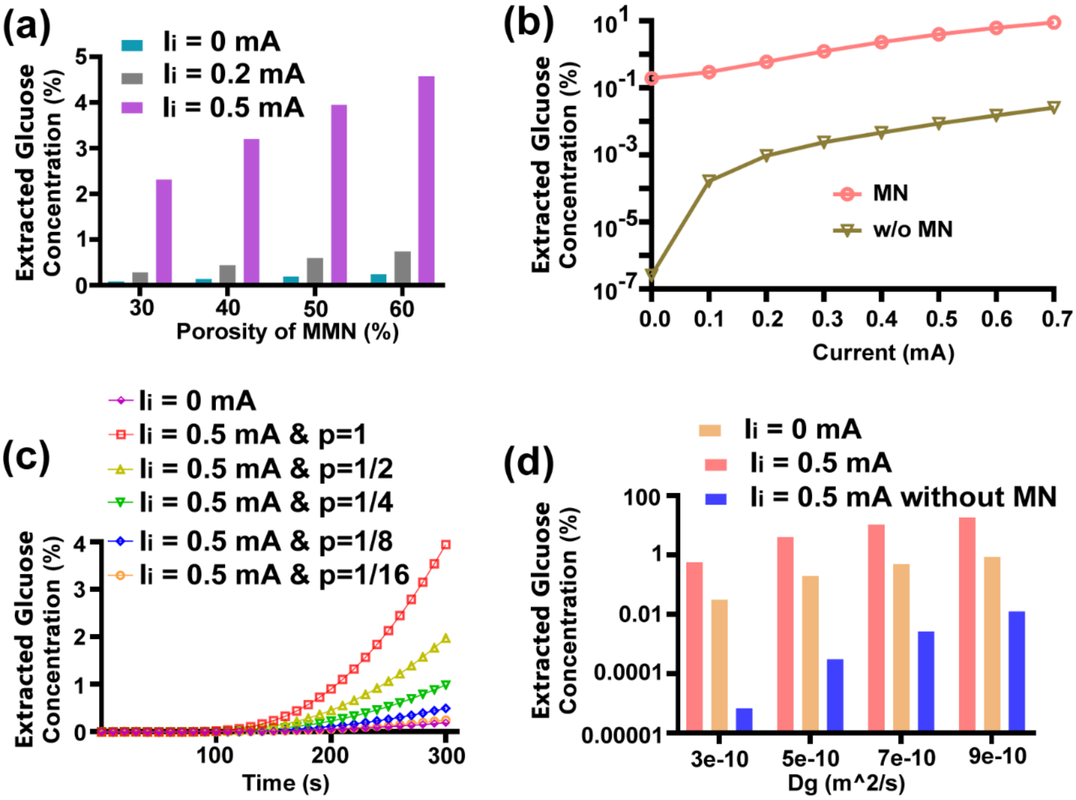

Figure S13. (a) Theoretical calculations of the effects of MN porosities (30%-60%) on glucose extraction, under different iontophoresis conditions. The extraction efficiency was improved with the porosity increased from 30% to 60%. (b) Theoretical calculations of the effects of electric filed on glucose extraction. (c) Uncharged glucose was extracted across skin layer into the sensing chamber. Na^+ and Cl^- underneath skin migrate toward the electrodes by the applied potentials. The iontophoresis of glucose is achieved by convective transport of glucose along with the ions. The electric charge of Na^+ is +1. The glucose moved to the cathode along with the Na^+. The glucose extraction results at different assumed convective ratios (from 1/16 to 1) were investigated. (d) The diffusion coefficient of small molecules in water is generally in the range of $3e^{-10}$ - $9e^{-10}$. The results using different diffusion coefficients (Dg) were also investigated.

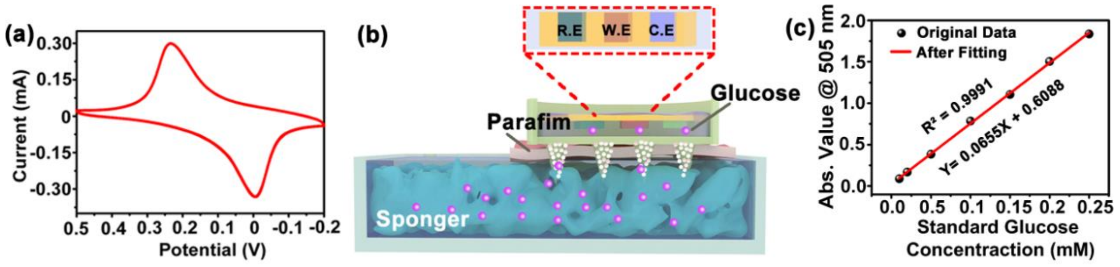

Figure S14. (a) The Cyclic Voltammetry curve of W.E./Cr/Au/PB electrode tested in KCl/HCl solution. The PB was deposited in situ on the W.E. by constant voltage 0.8 V electrodeposition 480 s in a fresh solution (100 mL) containing 2.5 mM $FeCl_3$, 100 mM KCl, 2.5 mM $K_3Fe(CN)_6$, and 100 mM HCl. The electrochemical activities potential of the W.E./Cr/Au/PB electrode were evaluated via cyclic voltammetry. The cyclic voltammetry at the KCl/HCl (0.1 M KCl and 0.01 M HCl) in DI solution were performed with a scan rate of 50 mV/s at the potential range of −0.2 to 0.5 V. The electrode exhibited a reduced potential at 0 V due to the successful deposition of PB on the electrode. (b) Schematic showing the experimental setup of RIMN sensor without iontophoresis (0 mA) for glucose sensing in vitro. The glucose in the bottom chamber diffuses freely through the MMN into the MN sensor's chamber at the back of the MN, where it is detected by the glucose electrode of MN sensor. (c) The relation of the optical absorbance (measured at 505 nm) with glucose concentrations. The relation was linearly fitted as a standard curve.

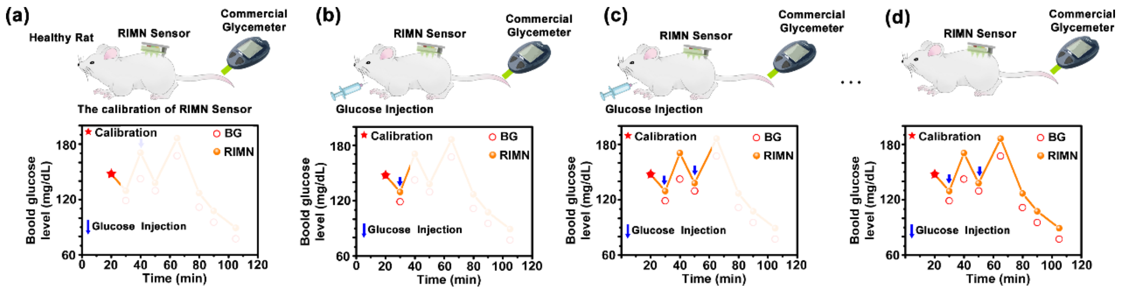

Figure S15. Illustration and detail analysis of the RIMN sensing experiment on the heathy rat model, related to Figure 3m in main text. (a) At t=0 min, the RIMN sensor was pressed against the back to penetrate the skin by MMN. Glucose sensing was performed every 10 min at the iontophoretic condition of 0.5 mA current and 300 s duration for each cycle. The detected current signal via RIMN sensor was converted to glucose concentration (the orange point). The actual BG were collected from the tail vein of the rats to measure BG by a standard Roche blood glucose meter (the red circle point). At t=20 min, the detected glucose level via RIMN was calibrated to the actual BG (indicated with asterisk). (b) At t=30 min, the rat was intraperitoneally injected with 5% glucose solution (indicated with blue arrow). The glucose level increased after glucose injection. (c) At t=50 min, the rat was intraperitoneally injected with 5% glucose solution again. The glucose level increased after glucose injection as well. (d) From t=70 min, the rising of glucose level was hindered due to effective BG regulation by healthy metabolic function.

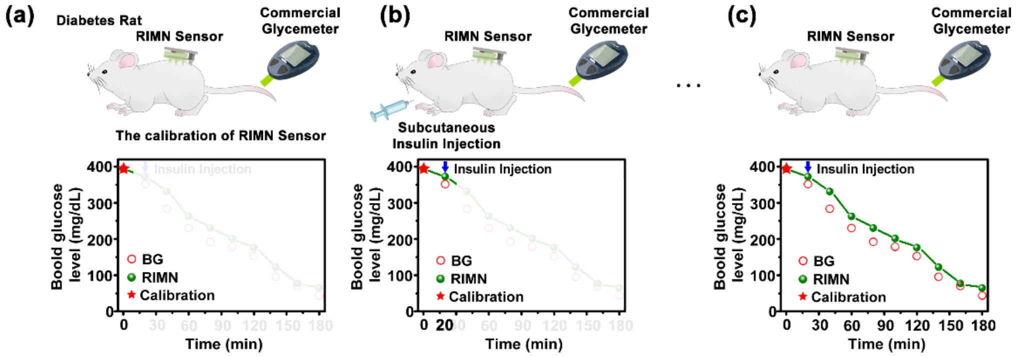

Figure S16. Illustration and detail analysis of the RIMN sensing experiment on the diabetic rat model, related to Figure 3n in main text. (a) At t=0 min, the RIMN sensor was pressed against the back to penetrate the skin by MMN. Glucose sensing was performed every 15 min at the iontophoretic condition of 0.5 mA current and 300 s duration for each cycle. The detected current signal via RIMN sensor was converted to glucose concentration (the orange point). The actual BG were collected from the tail vein of the rats to measure BG by a standard Roche blood glucose meter (the red circle point). At t=0 min, the detected glucose level via RIMN was calibrated to the actual BG (indicated with asterisk). (b) At t=20 min, the rat was subcutaneously injected with 5 IU insulin. The blue arrow indicated the time point of insulin injections. The glucose level gradually decreased to normoglycemia due to BG regulation by injected insulin.

Statistical analysis of detection accuracy

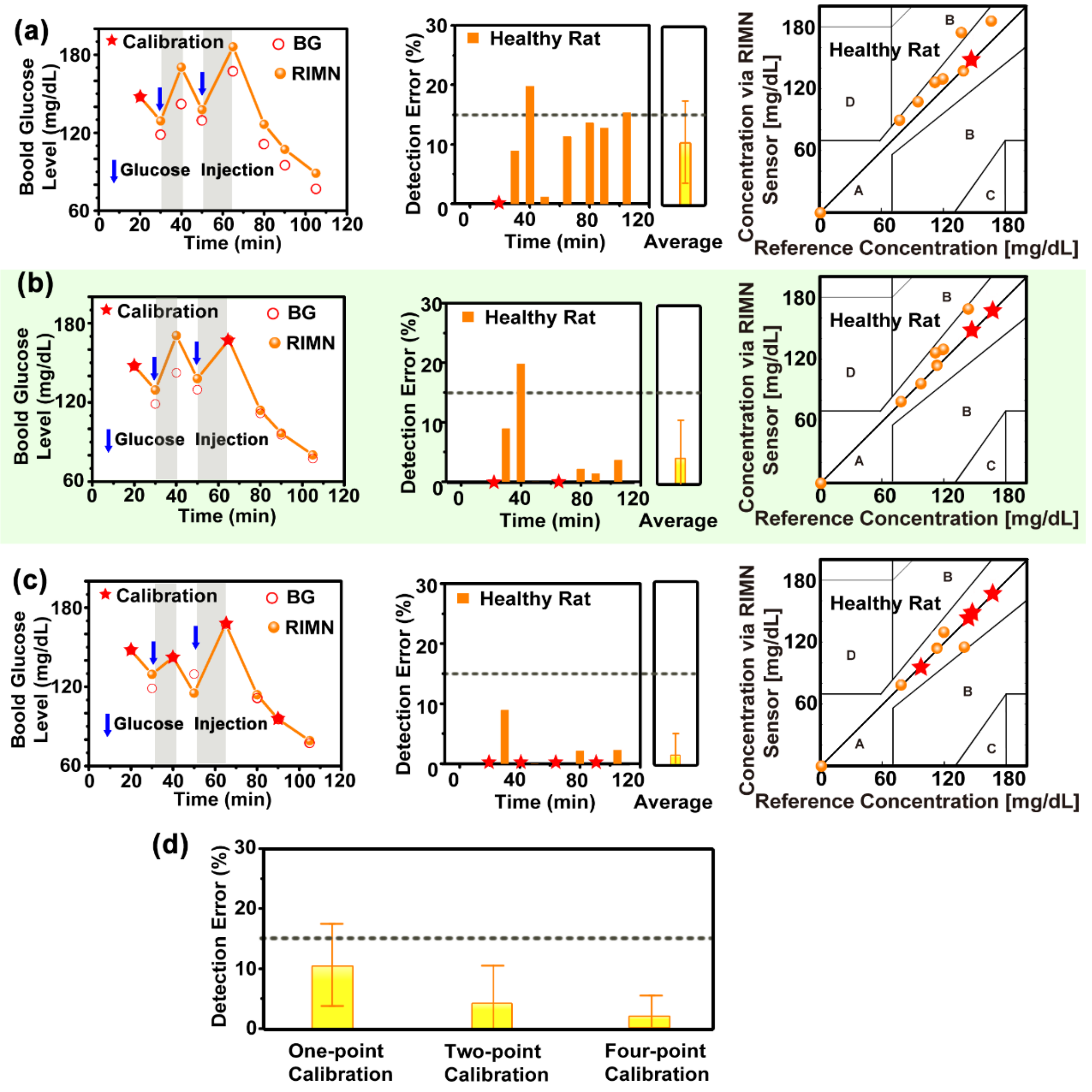

Figure S17. The RIMN sensing accuracies on healthy rats (related to Figure 3m in main text) using different times of BG calibrations. Left panel: the dynamic glucose signals recorded by RIMN sensors in vivo. Middle panel: statistical analysis showing the detection errors of RIMN sensor compared to the actual BG at corresponding time points. Right panel: the clarke’s error grid analysis showing the detection accuracy of RIMN sensor compared to the actual BG. The detected current signal via RIMN was converted to glucose concentration, and the actual BG were measured via standard glucose test strips. The blue arrow indicated the time point of glucose injections. The asterisks indicated RIMN signals calibrated to the actual BG. (a) Calibration using the first BG measurement (at time points t=20 min). The error of all the RIMN sensor-measured glucose signal was below 22%, with an average error of 10.4 ± 6.4%. In the clarke’s error grid analysis, 87.5% of data located in region A, corresponding to a sensing error <20% that is closely satisfying the clinical requirement of error <15%. (b) Calibration using two BG measurements (at time points t=20 and 70 min).

The error of all the RIMN sensor-measured glucose signal was below 19%, with an average error of 4.6 ± 6.3%. In the clarke's error grid analysis, 100% of located in region A. (c) Calibration using four BG measurements (at time points t=20, 40, 70, and 90 min). The error of all the RIMN sensor-measured glucose signal was below 17%, with an average error of 3.9 ± 5.9%. In the clarke's error grid analysis, 100% of data located in region A. (d) Statistical analysis of the average error using one, two, and four-points calibrations. One-point calibration could produce results closely satisfying the clinical requirement of error <15%. The uses of more calibration points could further improve the sensing accuracies, but induce more pain and inconvenience due to frequent BG measurements.

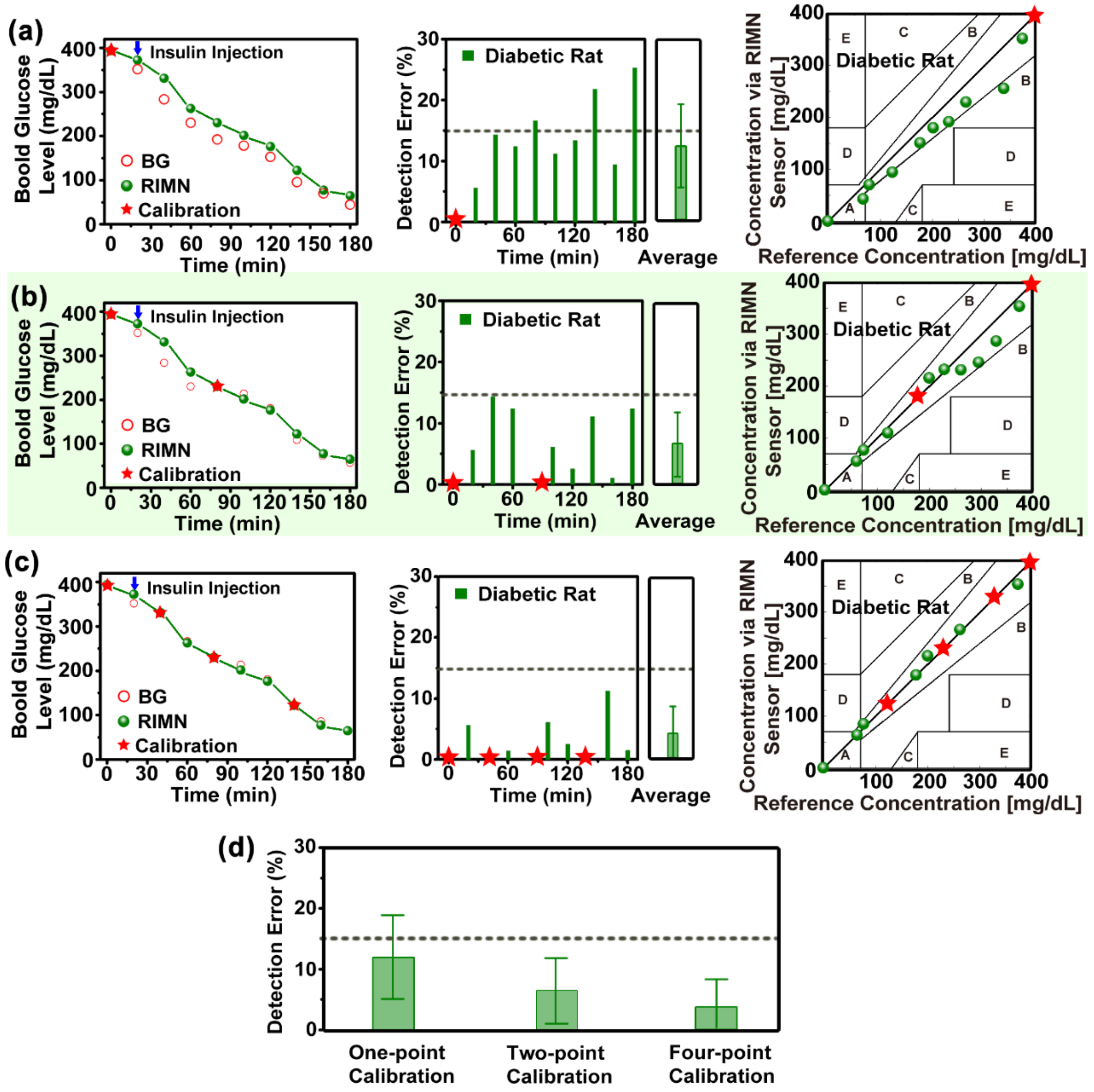

Figure S18. The RIMN sensing accuracies on diabetic rats (related to Figure 3n in main text) using different times of BG calibrations. Left panel: the dynamic glucose signals recorded by RIMN sensors in vivo. Middle panel: statistical analysis showing the detection errors of RIMN sensor compared to the actual BG at corresponding time points. Right panel: the clarke's error grid analysis showing the detection accuracy of RIMN sensor compared to the actual BG. The detected current signal via RIMN was converted to glucose concentration, and the actual BG were measured via standard glucose test strips. The blue arrow indicated the time point of insulin injections. The asterisks indicated RIMN signals calibrated to the actual BG. (a) Calibration using the first BG measurement (at time points t=0 min). The error of all the RIMN sensor-measured glucose signal was below 25%, with an average error of 12.9 ± 6.9%. In the clarke's error grid analysis, 90% of data located in region A, corresponding to an sensing error <20% that is closely satisfying the clinical requirement of error <15%. (b) Calibration using two BG measurements (at time points t=0 and 80 min). The error of all the RIMN sensor-measured glucose signal was below 14%, with an average error of 6.5 ± 5.3%. In the clarke's error grid analysis, 100% of data located in region A. (c) Calibration using four BG measurements (at time points t=20, 40, 70, and 90 min). The RIMN sensor in diabetic rat calibration of the four drop of blood (at time points t=0, 30, 80, and 140 min). The error of all the RIMN sensor-measured glucose signal was below 11%, with an average error of 2.8 ± 3.5%. In the clarke's error grid analysis, 100% of data located in region A. (d) Statistical analysis of the average error using one, two, and four-points calibrations. One-point calibration could produce results closely satisfying the clinical requirement of error <15%. The uses of more calibration points could further improve the sensing accuracy, but induced more pains and inconvenience due to frequent BG measurements.

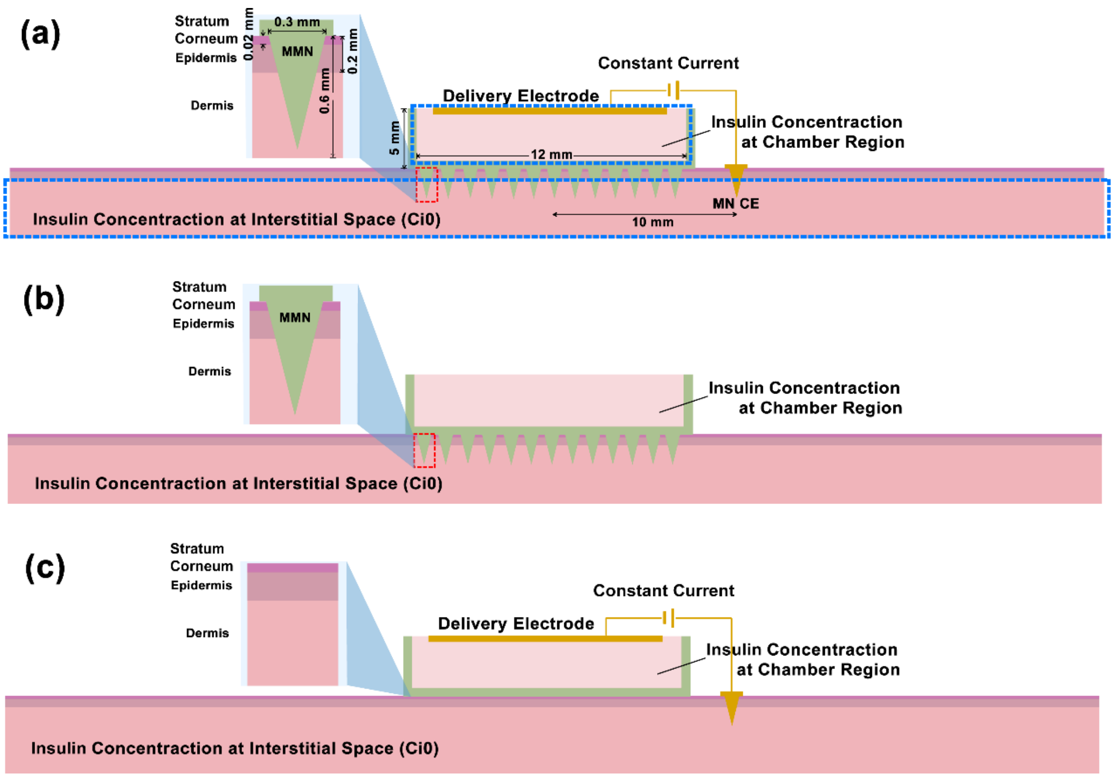

Figure S19. (a) Schematic of COMSOL simulation to calculate the electrical field and percutaneous potential on the IMN device and related detailed dimension parameters in the simulation. The geometry and setup were similar with the model of RIMN glucose extraction. Constant current was applied between the electrode and the MN CE. The top boundary of the chamber was set with influx of insulin concentration C_{i0}. The boundaries under skin were set open assuming the insulin could diffuse further to an unlimited space of body system. After releasing, the average insulin concentration in the interstitial space was calculated to evaluate the delivery efficiency. The released insulin amount was normalized by comparing to the initial insulin amount in the IMN device chamber. The average insulin concentrations in the area marked with blue dash lines were calculated, respectively. The average insulin concentration in the interstitial space was normalized to the average glucose concentration in the sensing chamber. (b) COMSOL 2D model of insulin delivery by MN device without iontophoresis. Insulin diffused from the delivery chamber to the interstitial space through the MMN. (c) COMSOL 2D model of insulin delivery by iontophoresis device without MMN. Constant current was applied between the electrode and the MN CE. Insulin was driven across skin layer into the interstitial space.

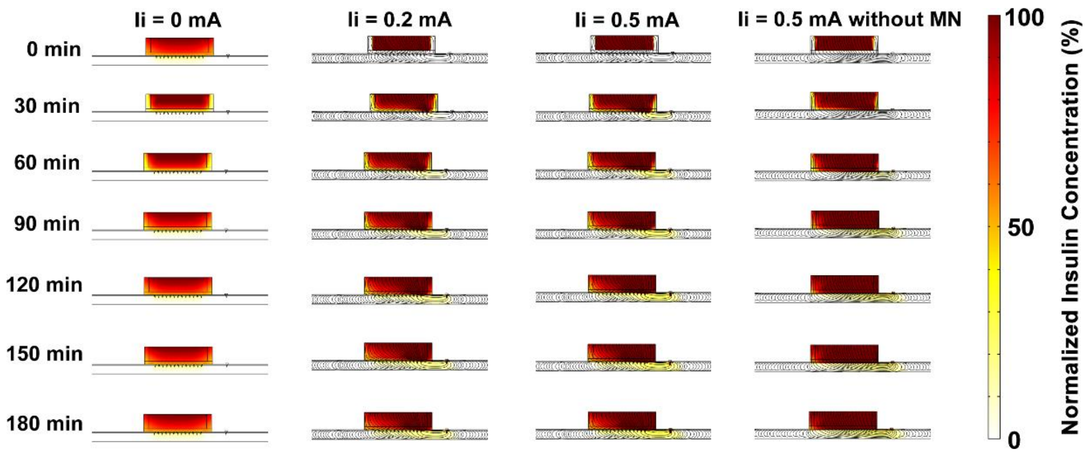

Figure S20. The electrical field distribution (indicated with black curves) and insulin concentration profile (indicated with brown color) at different time points (0-180 min) during iontophoresis with different current conditions (0-0.5 mA) via IMN device.

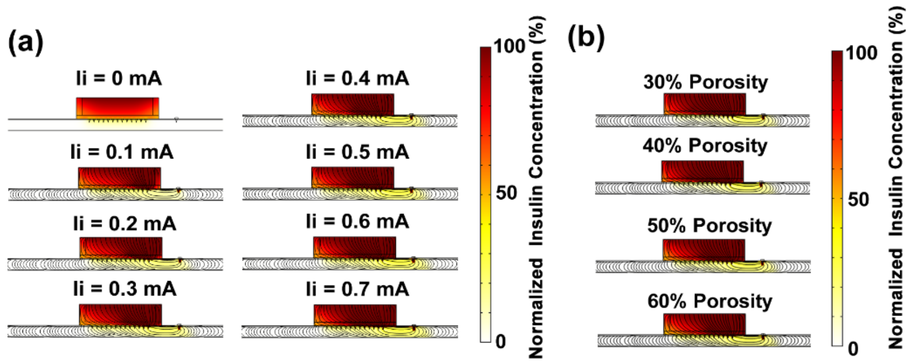

Figure S21. (a) The electrical field distribution (indicated with black curves) and insulin concentration profile (indicated with brown color) at t=180 s after iontophoresis at different current conditions (0-0.7 mA). (b) The electrical field distribution (indicated with black curves) and insulin concentration profile (indicated with brown color) at t=180 s using IMN device with different MMN porosities (30% - 60%).

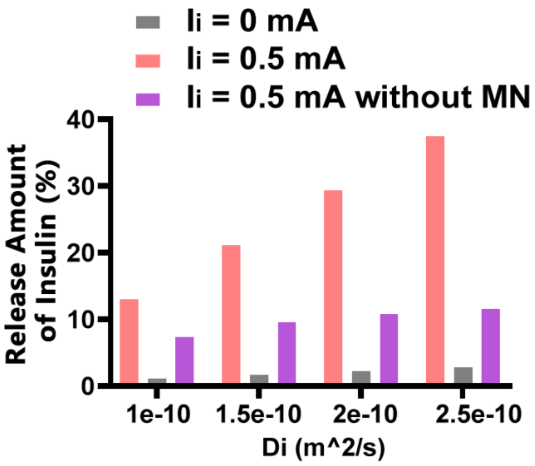

Fig S22. The diffusion coefficient of insulin in PBS is generally in the range of 1e^{-10} - 2.5e^{-10}. The results using different diffusion coefficients (Di) were investigated.

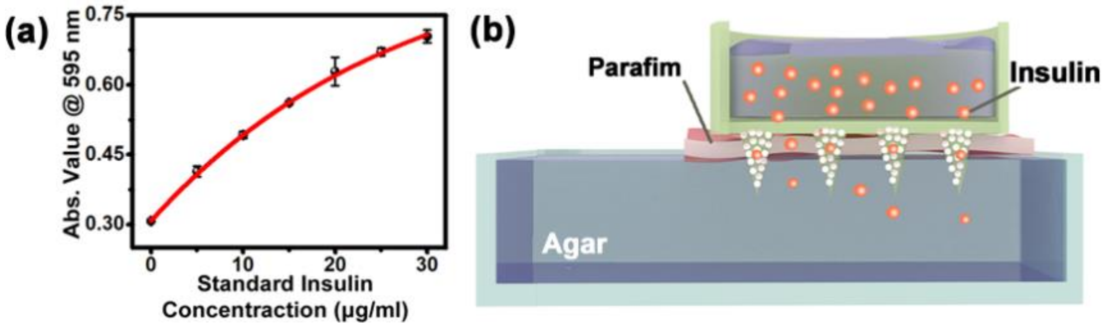

Figure S23. (a) The relation of the optical absorbance with the insulin concentration via Coomassie Plus protein assay. The absorbance was measured at 595 nm. The relation was linearly fitted as a standard curve. (b) Schematic showing the experimental setup of IMN device without iontophoresis for insulin delivery in vitro. Insulin in the IMN device's chamber was freely diffused through the MMN into the bottom chamber to be collected. The amount of collected insulin was detected using a Coomassie Plus protein assay.

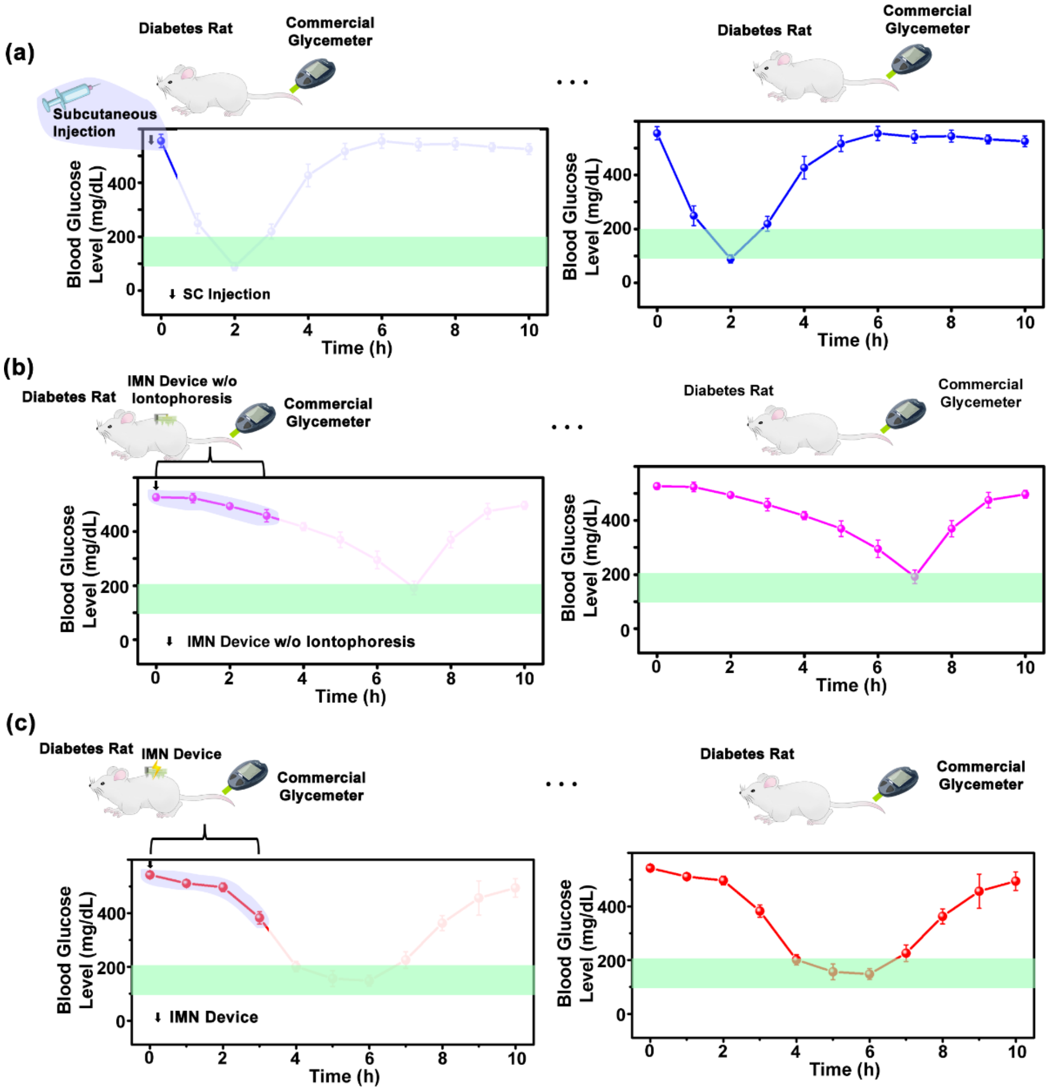

Figure S24. Illustration and detail analysis of the IMN device delivery experiments, related to Figure 4k in main text. The BG fluctuation were continuously monitored for 10 h. (a) Subcutaneous injection of 5 UI insulin. The black arrow indicated the time point (t=0 h) of insulin subcutaneous injection. The BG of diabetic rats was rapidly reduced to the lowest value of 100 mg/dL within two hours. Subsequently, the BG rapidly rebounded to hyperglycemia after two hours. (b) Application of MN delivery without iontophoresis. The black arrow indicated the time point (t=0 h) of MN delivery without iontophoresis, and the delivery lasted for 3 hours. BG slowly decreased to normoglycemic at t=7 h, followed by a rapid rebound to hyperglycemia over the next 3 hours. (c) Application of IMN device with iontophoresis at 0.5 mA. The black arrow indicated the time point (t=0 h) of IMN device without iontophoresis, and the device application lasted for 3 hours. BG decreased to normoglycemia at t=4 h, and remained stable for 3.3 hours.

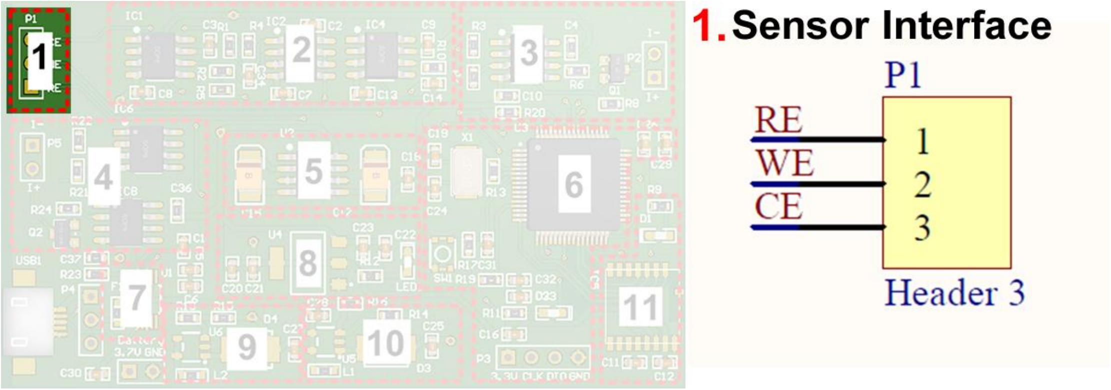

Figure S25. Circuit diagram showing the sensor interface (Circuit Component#1) of the IWCS. The electrical signal output by the sensor is connected to the interface through wire, and the electrical signal carrying glucose concentration information will enter the glucose signal conditioning circuit for further processing.

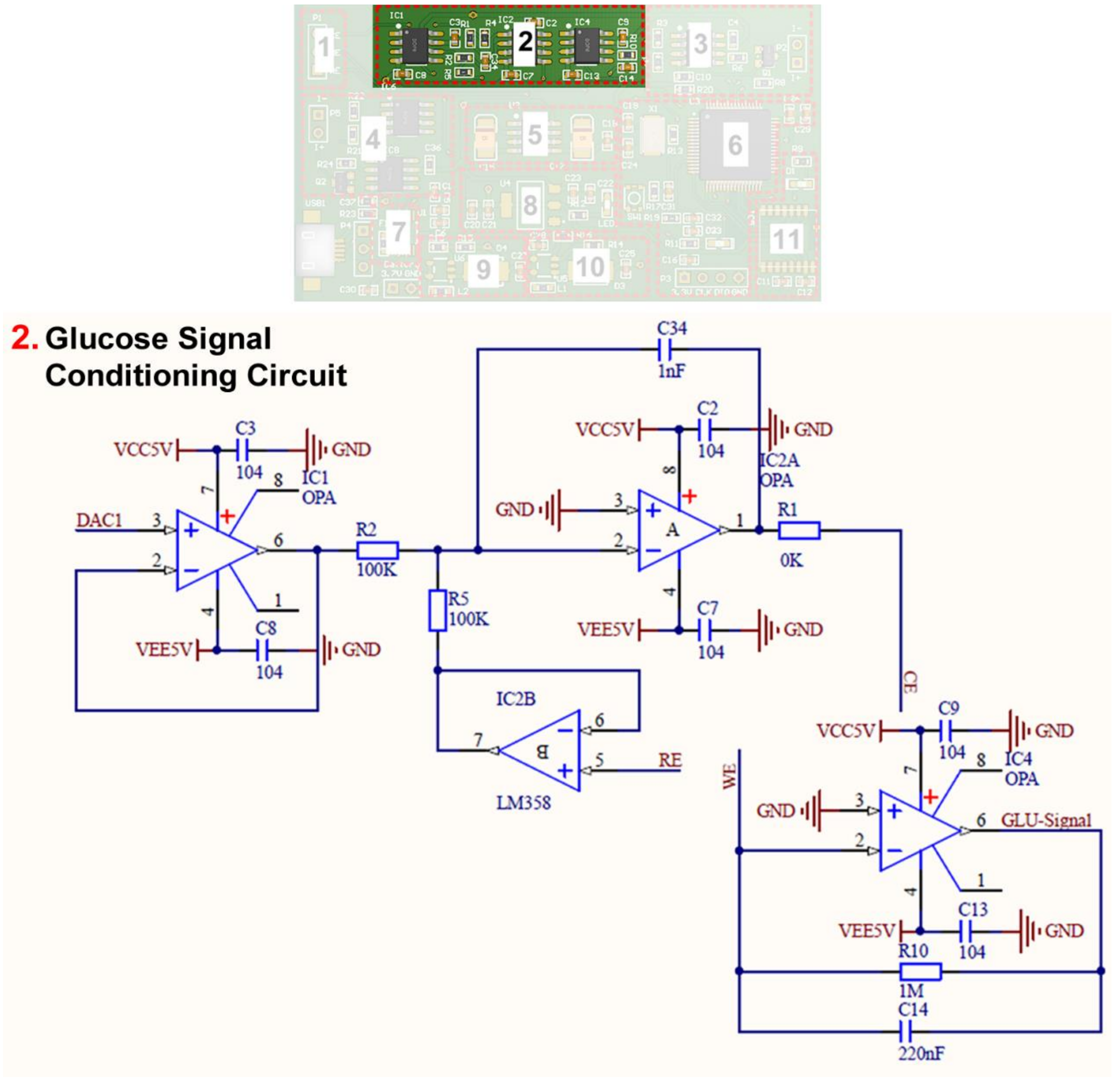

Figure S26. Circuit diagram showing the glucose signal conditioning circuit (Circuit Component#2) of the IWCS. The glucose signal conditioning circuit was consisted of control

amplifier, reverse follower and transimpedance amplifier. The potential on the reference electrode was controlled by the input voltage DAC1 because R2 was equal to R5. The working electrode was connected to the reverse input end of the transimpedance amplifier. The current on the working electrode I was converted to a voltage output by the transimpedance amplifier and the voltage output equal $I*R_{10}$.

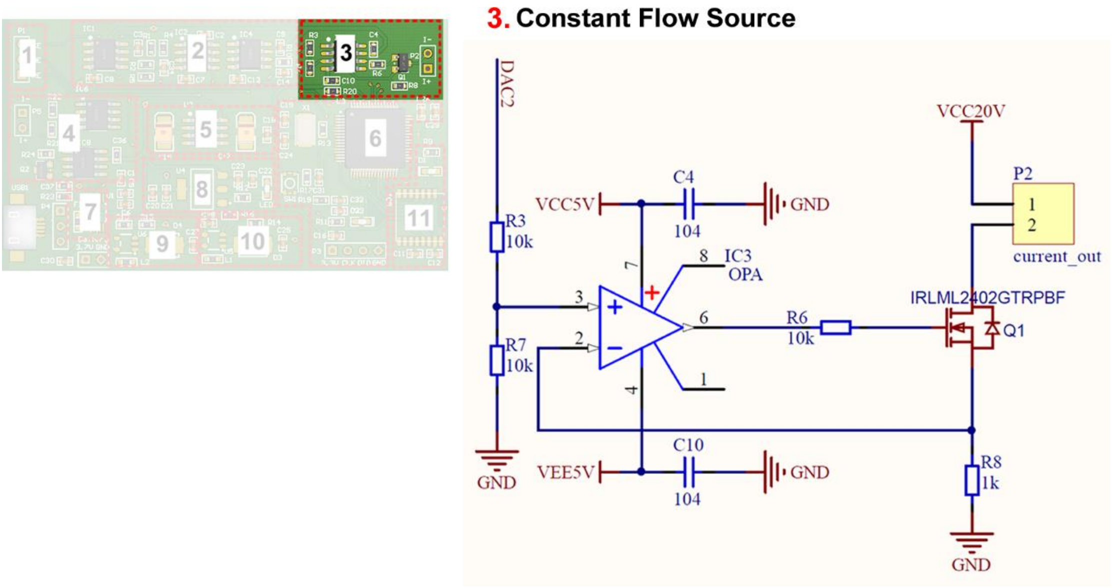

Figure S27. Circuit diagram showing the constant flow source for glucose extraction (Circuit Component#3) of the IWCS. The constant current source circuit was mainly composed of input stage and output stage. The input stage provided the constant voltage DAC2 and the transistor IRLML2402GTRPBF output a required constant current for glucose extraction.

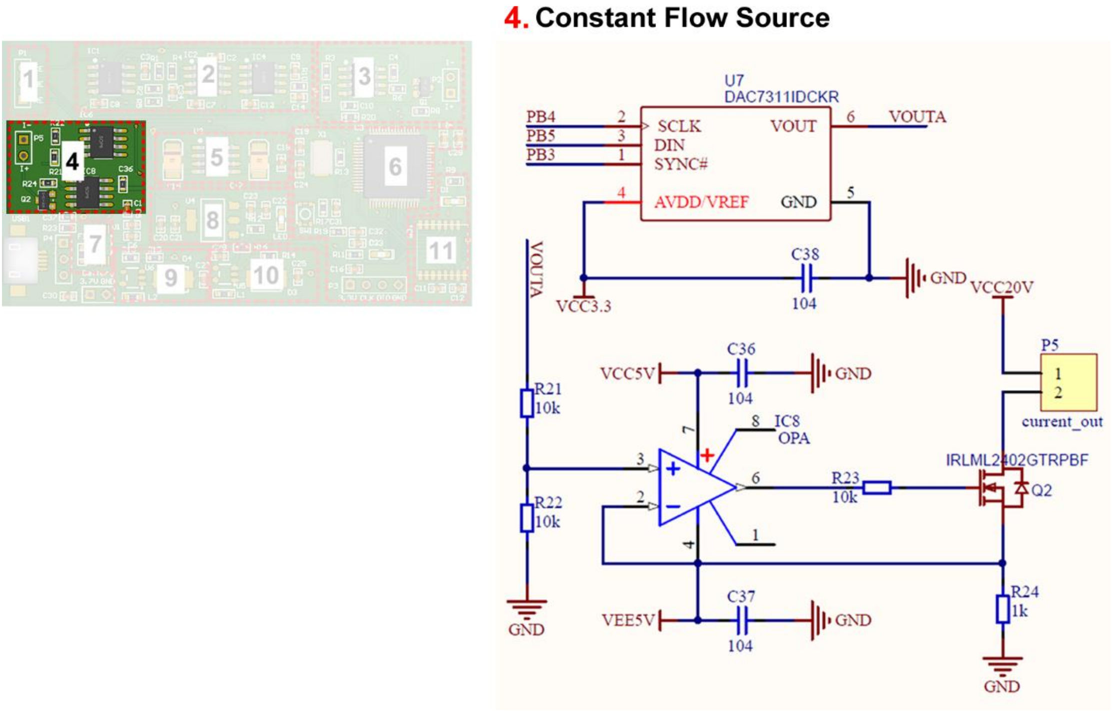

Figure S28. Circuit diagram showing the constant flow source for drug delivery (Circuit Component#4) of the IWCS. This constant flow source circuit was consisted of a constant flow transfer circuit and a digital-to-analog transfer circuit. The digital-to-analog converter DAC7311IDCKR output a constant voltage by the control of the STM32 minimum system. The constant voltage performed as the input stage of the constant flow transfer circuit and the transistor output a required constant current for drug delivery.

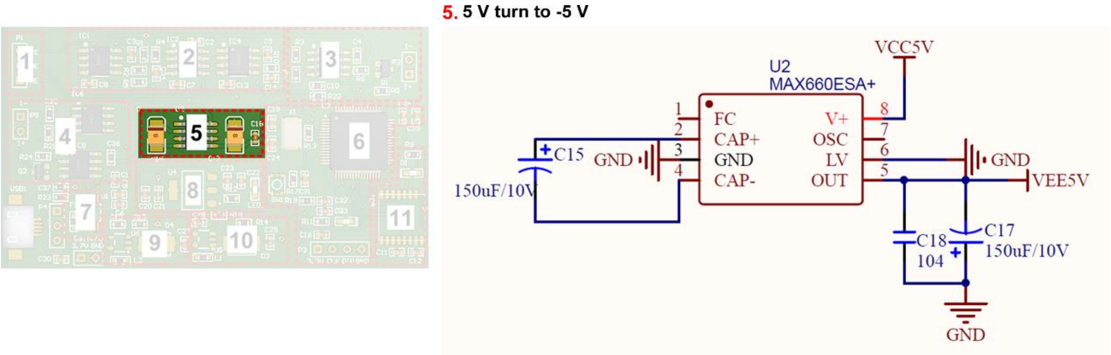

Figure S29. Circuit diagram showing the 5 V to -5 V voltage conversion circuit (Circuit Component#5) in the IWCS. The voltage conversion circuit transferred 5 V into -5 V by the CMOS monolithic voltage converter MAX660ESA+. The converter output negative voltage at pin5. Negative voltage provided power support for operational amplifiers in the glucose conditioning circuit.

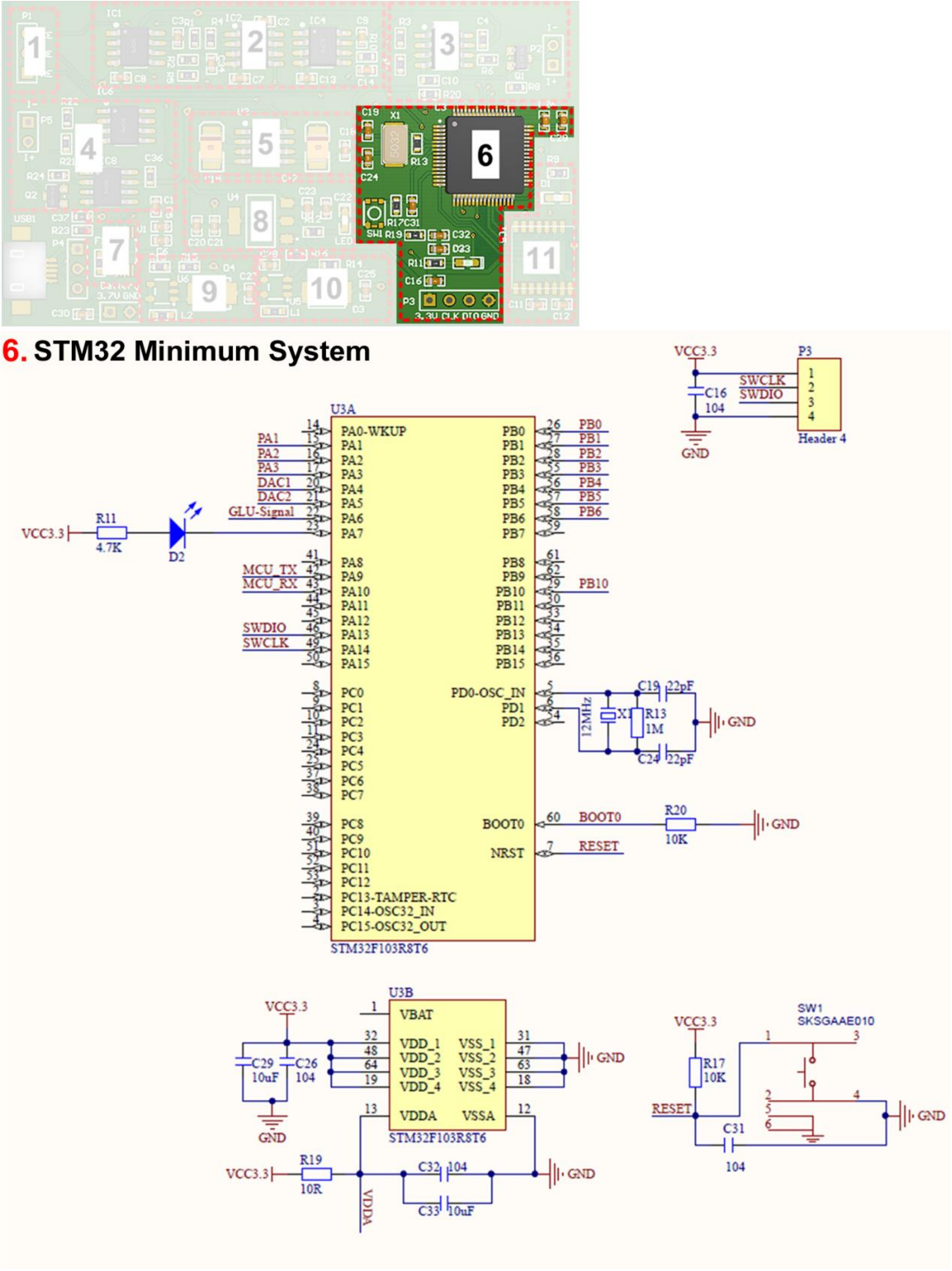

Figure S30. Circuit diagram showing the STM32 Minimum System (Circuit Component#6) in the IWCS. The STM32 minimum system was mainly consisted of power supply (Supplying working power), clock circuit, debugging interface, reset circuit, and MCU. The STM32 minimum system provided constant voltage to the glucose conditioning circuit and the constant flow source circuit. It controlled the digital-to-analog converter through command. The glucose signal was stored and processed by this system, and then sent the data through the Bluetooth section.

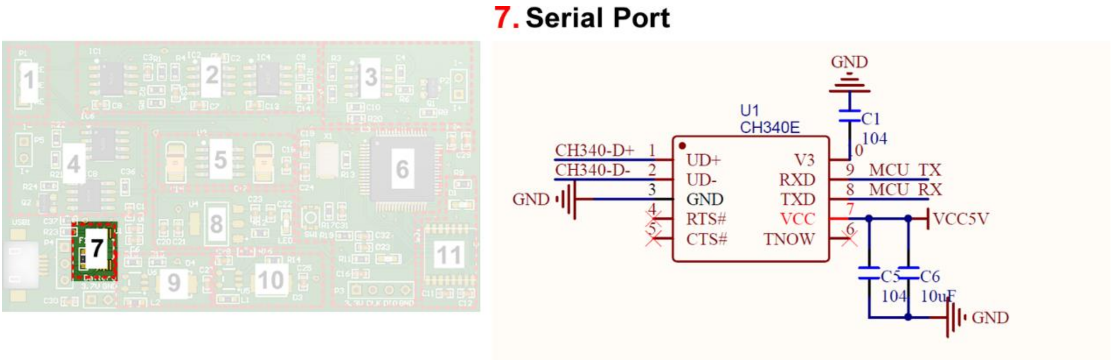

Figure S31. Circuit diagram showing the serial port (Circuit Component#7) of IWCS. The serial port circuit transferred USB to serial port by the USB bus transfer chip CH340E. This circuit converted the parallel data characters received from MCU into continuous serial data stream and send them out. At the same time, it converted the serial data stream received into parallel data characters to MCU.

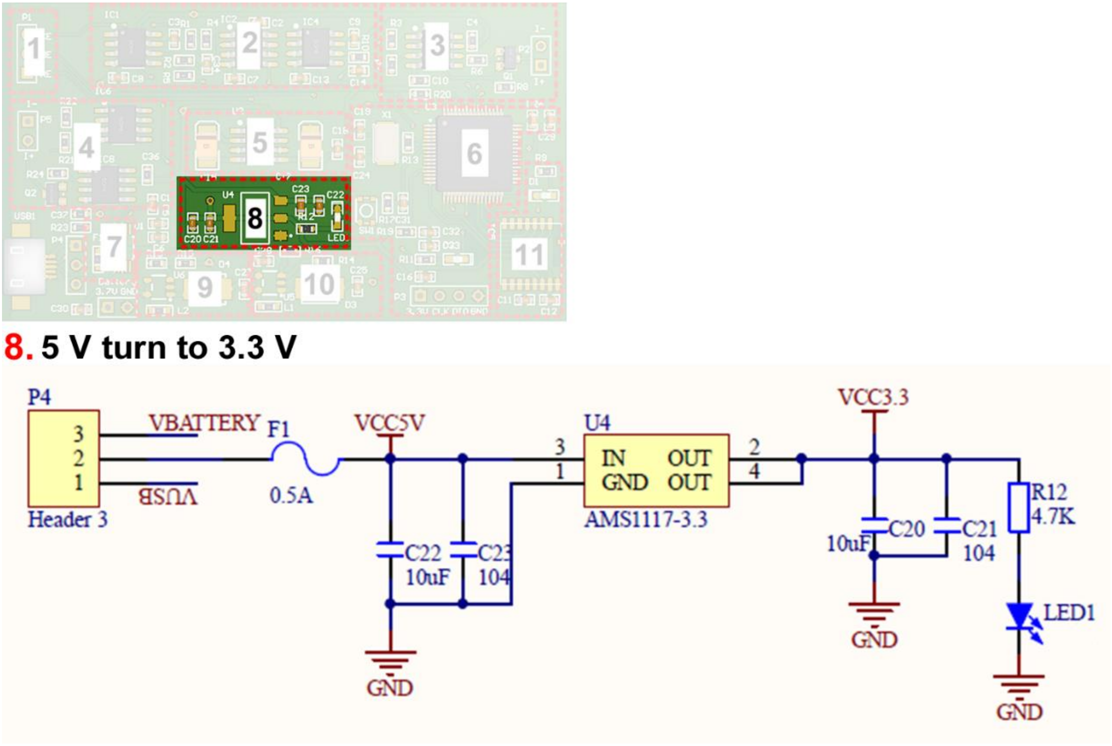

Figure S32. Circuit diagram showing the 5 V to 3.3 V voltage conversion circuit (Circuit Component#8) in the IWCS. The voltage conversion circuit transferred 5 V to 3.3 V by the voltage regulator AMS1117. The converter output 3.3 V voltage at pin2. The 3.3 V-voltage provided power support for the minimum STM32 system and the Bluetooth section.

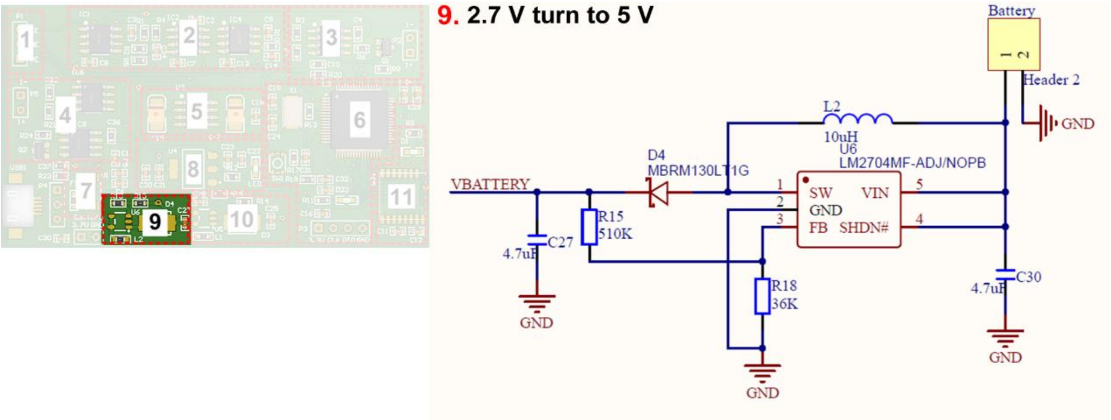

Figure S33. Circuit diagram showing the 2.7 V to 5 V voltage conversion circuit (Circuit Component#9) of the IWCS. External device input 2.7 V voltage signal through the USB. This voltage conversion circuit transferred the 2.7 V into 5 V by the boost converter LM2704MF-ADJ/NOPB. The 5 V-voltage provides support for operational amplifiers in the glucose conditioning circuit.

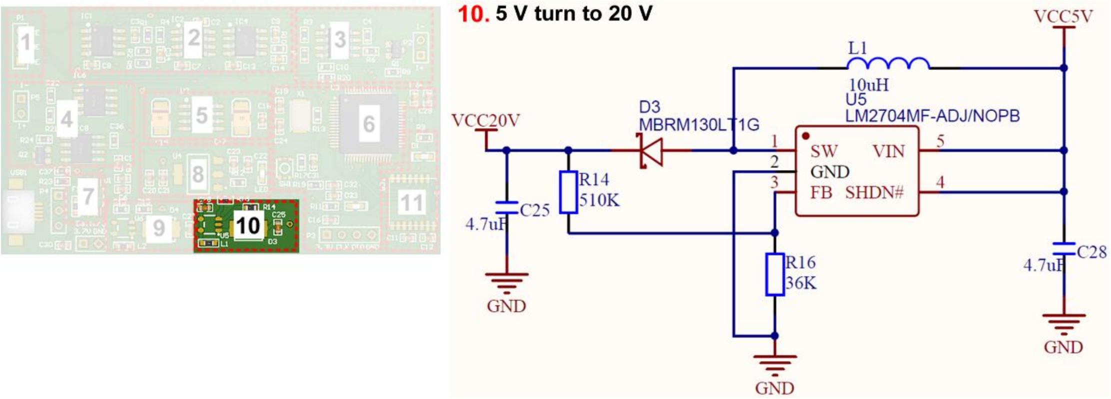

Figure S34. Circuit diagram showing the 5V turn to 20V voltage conversion circuit (Circuit Component#10) of the IWCS. The boost converter LM2704MF-ADJ/NOPB could provide 20 volts maximum output voltage. The 5 V power was enhanced to 20 V by the voltage conversion circuit, and the 20 volts supplies power was used to driven the constant flow source.

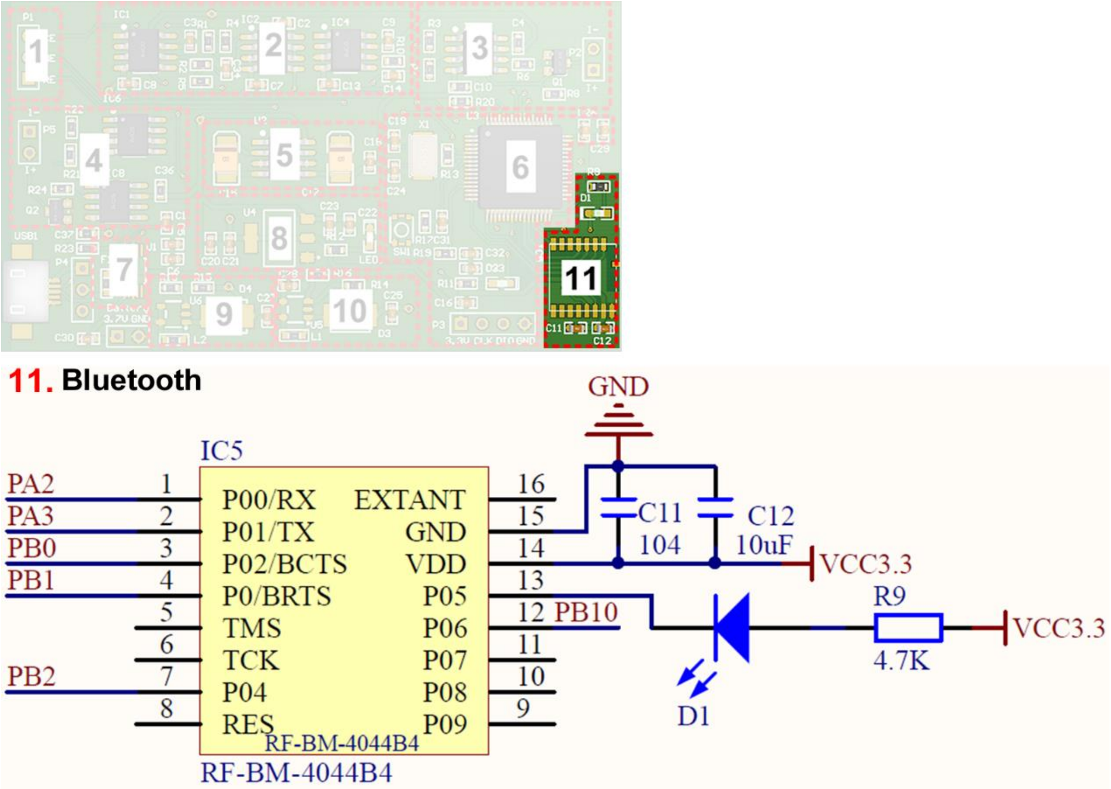

Figure S35. Circuit diagram showing the Bluetooth interface (Circuit Component#11) of the IWCS. The Bluetooth realized wireless data transmission and reception between the IWCS and the mobile phone. The Bluetooth section sent the glucose concentration signal stored in the STM32 minimum system to the mobile device, displaying the glucose concentration data in real-time.

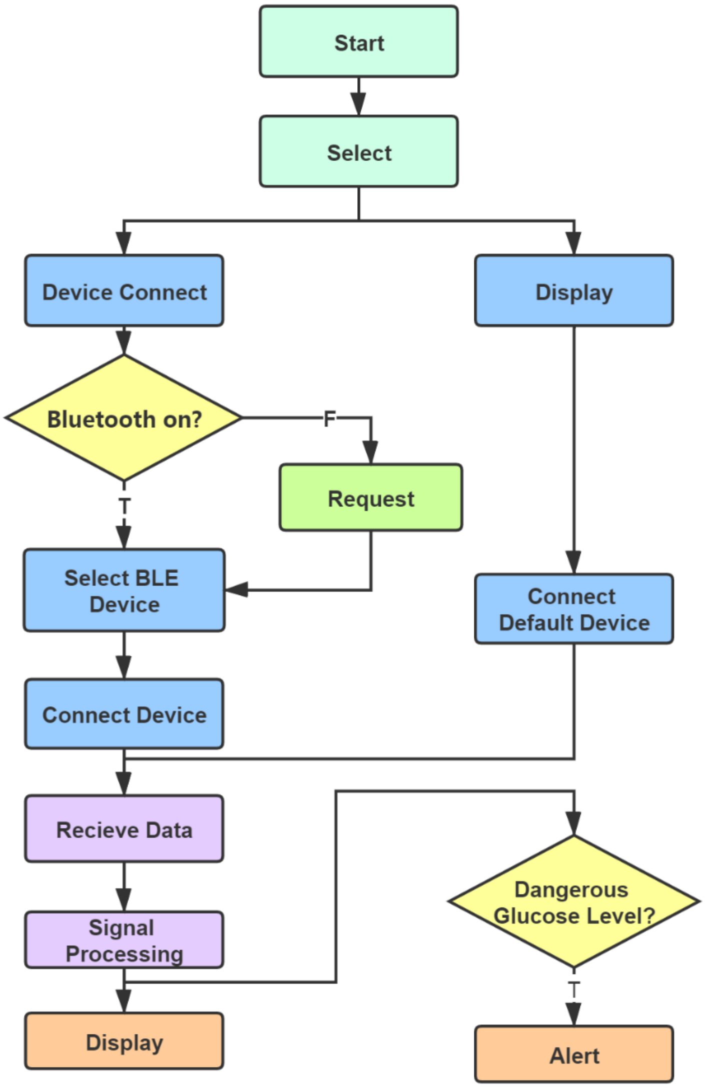

Figure S36. The flow chart of user interface design of the IWCS. To provide the user-friendly interface for glucose data collection, a mobile application was designed to support the IWCS. At the beginning of the program, user need to connect IWCS's Bluetooth manually. Application received and resolved the stream of data that transmitted in real time from the IWCS. Since IWCS did not filter the analog signal, it was necessary to filter the data before calculating the glucose concentration value to eliminate noise interference. The glucose concentration signal was a slowly changing signal, close to a direct-current signal. Therefore, the Butterworth low-pass filter with the cut-off frequency of 1 Hz was adopted to filter the glucose concentration signal. The output data of the digital filter was converted to glucose concentration value and displayed on the interface in real time, and the user can also see the previous history of blood glucose value by sliding the display interface. Moreover, user can set the threshold of glucose concentration value related to hyperglycemia to alert the user by alarm. More functions of signal calculation into glucose concentration, BG calibration and

triggering delivery could be further developed in the APP.

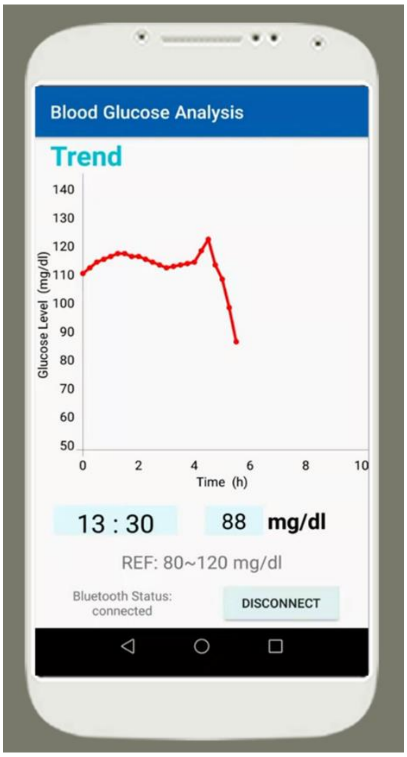

Figure S37. The photograph showing the detail interface of the APP design on smartphone. The APP could be further developed. More functions of signal calculation into glucose concentration, BG calibration and triggering delivery could be integrated into the APP.

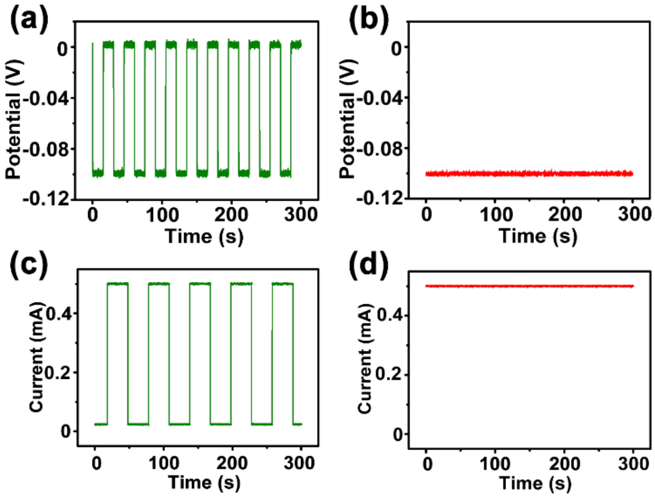

Figure S38. Detail discussion of the tests of circuit functions, related to Figure 5e and 5f in main text. (a) The voltage output of intermittent mode by the as-prepared FPCB. The DAC of the MCU output a rectangular wave which has 100 mV amplitude, 50% duty radio and 60 s period. (b) The voltage output of continuous mode by the as-prepared FPCB. The DAC of the MCU output a 100 mV DC signal. (c) The current output of intermittent mode by the as-prepared FPCB. A rectangular wave which has 500 mA amplitude, 50% duty radio and 60 s

period was output. (d) The current output of continuous mode by the as-prepared FPCB. A DC signal of 500 mA amplitude was output.

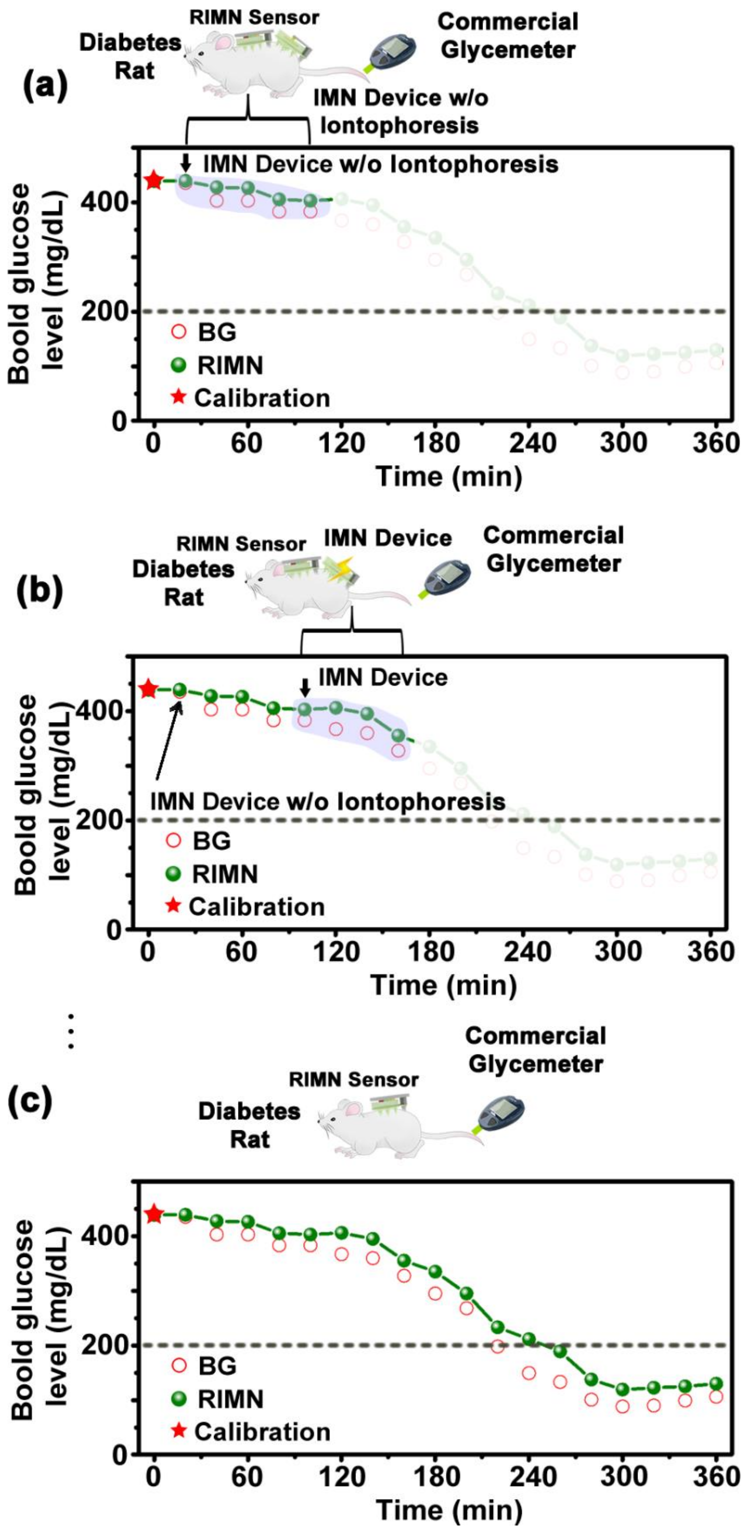

Figure S39. Illustration and detail analysis of simultaneous application of RIMN Sensor and IMN device on diabetic rat model, related to Figure 5k in main text. (a) At t=0 min, the RIMN

sensor and IMN devices were both pressed against the rat's back to penetrate the skin by MMN. Glucose sensing by RIMN sensor was performed every 20 min at the iontophoretic condition of 0.5 mA current and 300 s duration for each cycle, while standard glucose test strips were employed to measure rat's BG. The detected current signal via RIMN sensor was converted to glucose concentration (the orange point). The actual BG were collected from the tail vein of the rats to measure BG by a standard Roche blood glucose meter (the red circle point).

At t=0 min, the detected glucose level via RIMN was calibrated to the actual BG (indicated with asterisk). The diabetic rats were administered by MN without iontophoresis for the initial 80 min, and glucose level was observed to remain at the hyperglycemic state. (b) At t=80 min, MN-iontophoretic treatment was applied for 60 min, and the BG gradually decreased. (c) The BG decreased significantly to normoglycemia within 1.5 h and then remained stable. The black arrow indicated the time point of insulin delivery by IMN device. The dash line indicated the boundary of normoglycemia (BG<200 mg/dL).

Statistical analysis of detection accuracy

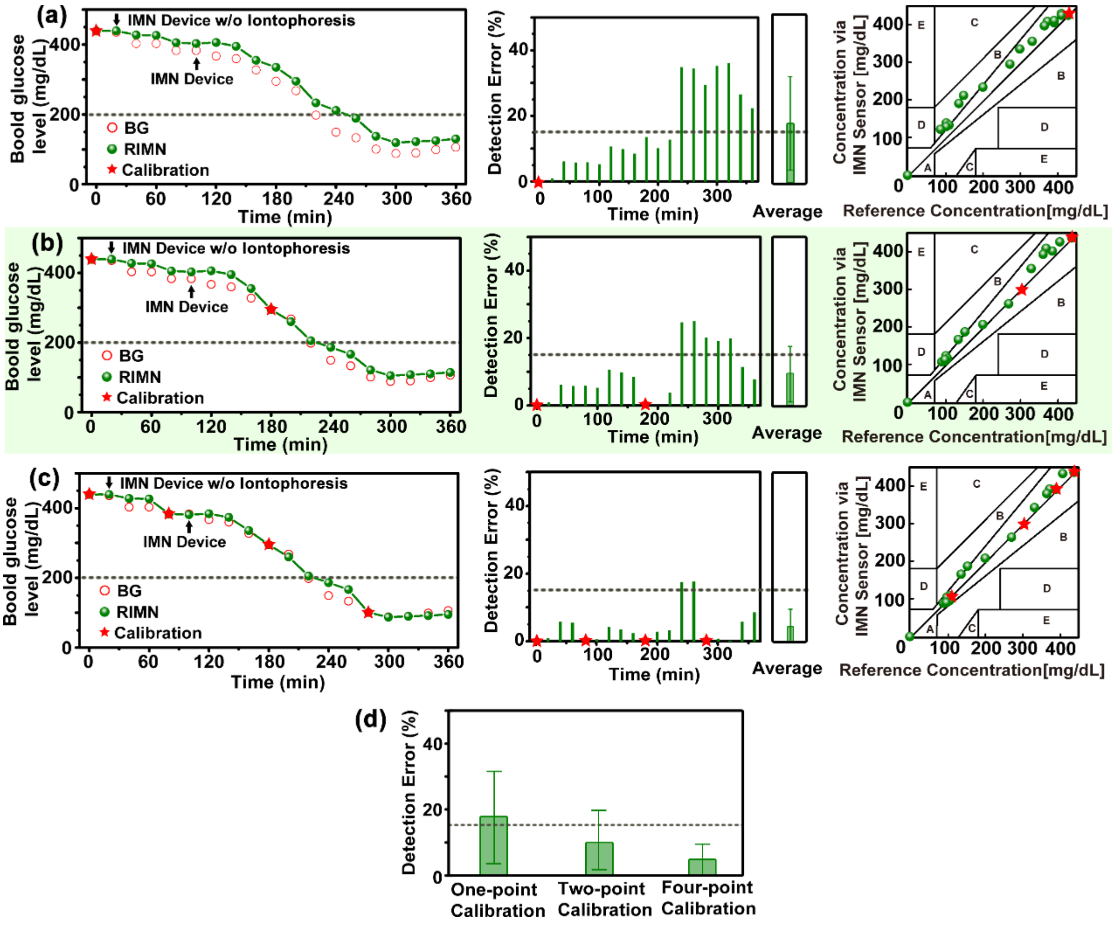

Figure S40. (a) Simultaneous application of RIMN Sensor and IMN device on diabetic rat model (related to Figure 5k in main text) using different times of BG calibrations. Left panel: the dynamic glucose signals recorded by RIMN sensors in vivo. Middle panel: statistical analysis showing the detection errors of RIMN sensor compared to the actual BG at corresponding time points. Right panel: the clarke's error grid analysis showing the detection accuracy of RIMN sensor compared to the actual BG. The detected current signal via RIMN was converted to glucose concentration, and the actual BG were measured via standard glucose test strips. The black arrow indicated the stating time point of insulin delivery by IMN device with iontophoresis. The asterisks indicated RIMN signals calibrated to the actual BG. (a) Calibration using the first BG measurement (at time points t=0 min). The error of all the RIMN sensor-measured glucose signal was below 40%, with an average error of 17.5 ± 13.9%. In the clarke's error grid analysis, 90% of data located in region A. (b) Calibration using two BG measurements (at time points t=0 and 180 min). The error of all the RIMN sensor-measured glucose signal was below 25%, with an average error of 9.5 ± 8.3%. In the clarke error grid analysis, 89% of data located in region A. (c) Calibration using four BG measurements (at time points t=0, 80, 180, and 280 min). The error of all the RIMN sensor-measured glucose signal was below 17%, with an average error of 4.13 ± 5.18%. In the clarke error grid analysis, 100% of healthy group data located in region A. (d) Statistical analysis of the average error using one, two, and four-points calibrations. One-point calibration could produce results closely satisfying the clinical requirement of error <15%. The uses of more calibration points could further improve the sensing accuracy, but induced more pains and inconvenience due to frequent BG measurements.

References:

[1] N. Pavselj, D. Miklavcic, *IEEE Transactions on Biomedical Engineering* **2008**, 55, 1927.

[2] J. Feng, J. Mo, A. Zhang, D. Liu, L. Zhou, T. Hang, C. Yang, Q. Wu, D. Xia, R. Wen, J. Yang, Y. Feng, Y. Huang, N. Hu, G. He, X. Xie, *Nanoscale* **2020**, 12, 5103.

[3] A. P. R. A, S. C. M. A, X. C. A, G. J. P. F. A, C. F. A, M. A. F. K. A. B, *Journal of Controlled Release* **2013**, 166, 87.

[4] R. Schulz, K. Yamamoto, A. Klossek, R. Flesch, S. Honzke, F. Rancan, A. Vogt, U. Blume-Peytavi, S. Hedtrich, M. Schafer-Korting, E. Ruhl, R. R. Netz, *Proceedings of the National Academy of Sciences of the United States of America* **2017**, 114, 3631.

[5] O. Gursky, J. Badger, Y. Li, D. L. Caspar, *Biophysical Journal* **1992**, 63, 1210.

[6] C. Jeworrek, O. Hollmann, R. Steitz, R. Winter, C. Czeslik, *Biophysical Journal* **2009**, 96, 1115.
